# Supplementary material for: Mast cell granule motility and exocytosis is driven by dynamic microtubule formation and kinesin-1 motor function
Source: PLoS One. 2022 Mar 22;17(3):e0265122. doi: 10.1371/journal.pone.0265122 (PMC8939832; doi:10.1371/journal.pone.0265122)
Supplement: S1 Fig — Confocal images from live-cell imaging of microtubule dynamics and granule movement of mast cells treated with 100 μM kinesore (see S6 Video via https://doi.org/10.6084/m9.figshare.19349573.v1). RBL-2H3 cells were transfected with EB3-tdTomato to label nascent microtubules and incubated with Lysotracker green to label granules. Cells were imaged for 1 min, then antigen-stimulated and concurrently kinesore was added, followed by 15 min of imaging. Images were extracted from the green channel at 5, 10 and 15 min time points. The intensity threshold was set to 1% and particles between 4–50 square pixels were counted in ImageJ. (PDF) [file pone.0265122.s001.pdf]

S1 Fig. Supporting Information

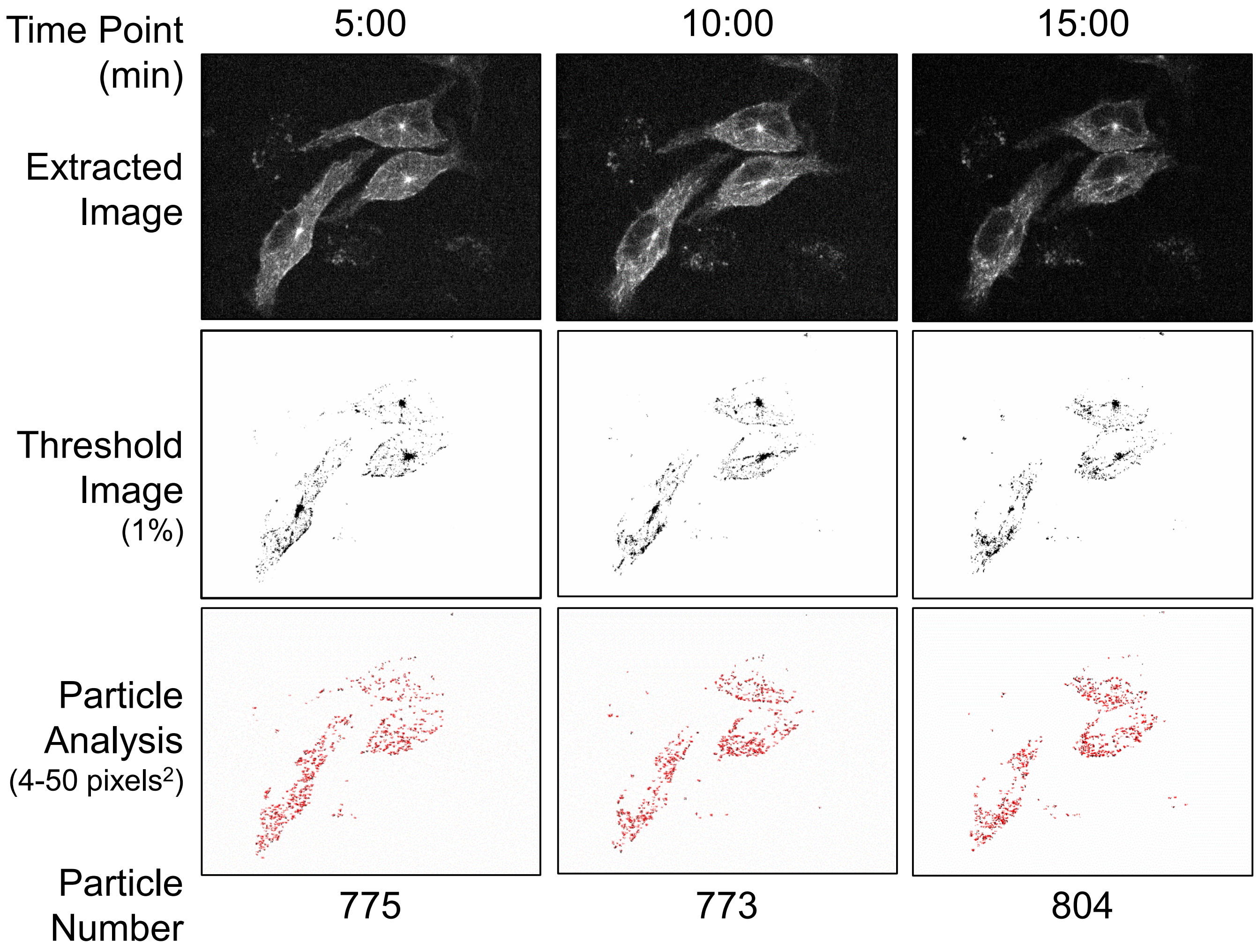

S1 Fig. Analysis of EB3-puncta after kinesore treatment.
Confocal images from live-cell imaging of microtubule dynamics and granule movement of mast cells treated with 100 mM kinesore (*see Movie 6*). RBL-2H3 cells were transfected with EB3-tdTomato to label nascent microtubules and incubated with Lysotracker green to label granules. Cells were imaged for 1 min, then antigen-stimulated and concurrently 100 mM kinesore was added, followed by 15 min of imaging. Images were extracted from the green channel at 5, 10 and 15 min time points. The intensity threshold was set to 1% and particles between 4-50 square pixels were counted in ImageJ.
