# Supplementary material for: Mast cell granule motility and exocytosis is driven by dynamic microtubule formation and kinesin-1 motor function
Source: PLoS One. 2022 Mar 22;17(3):e0265122. doi: 10.1371/journal.pone.0265122 (PMC8939832; doi:10.1371/journal.pone.0265122)
Supplement: S2 Fig — Live RBL-2H3 cells were labelled with LysoTracker Red (ThermoFisher). Cells were then left unstimulated (A), or antigen-stimulated for 20 min (B). Cells were fixed and CD63-positives vesicles were immuno-labelled with monoclonal CD63 antibodies (clone AD1, BioRad). F-actin was labelled with phalloidin iFluor-405 (Abcam). Images were taken with a Zeiss Observer Z1 epifluorescence microscope using a 63X 1.4 NA objective. Bottom panels show zoomed images of area indicated in the upper panels. Note that CD63 antibodies label many outlier vesicles that are not labelled with LysoTracker Red (arrows), while LysoTracker Red labelled vesicles predominately overlap with CD63 labelling. Scale bar, 10 μm. (PDF) [file pone.0265122.s002.pdf]

S2 Fig. Supporting Information

A Unstimulated RBL-2H3 cells

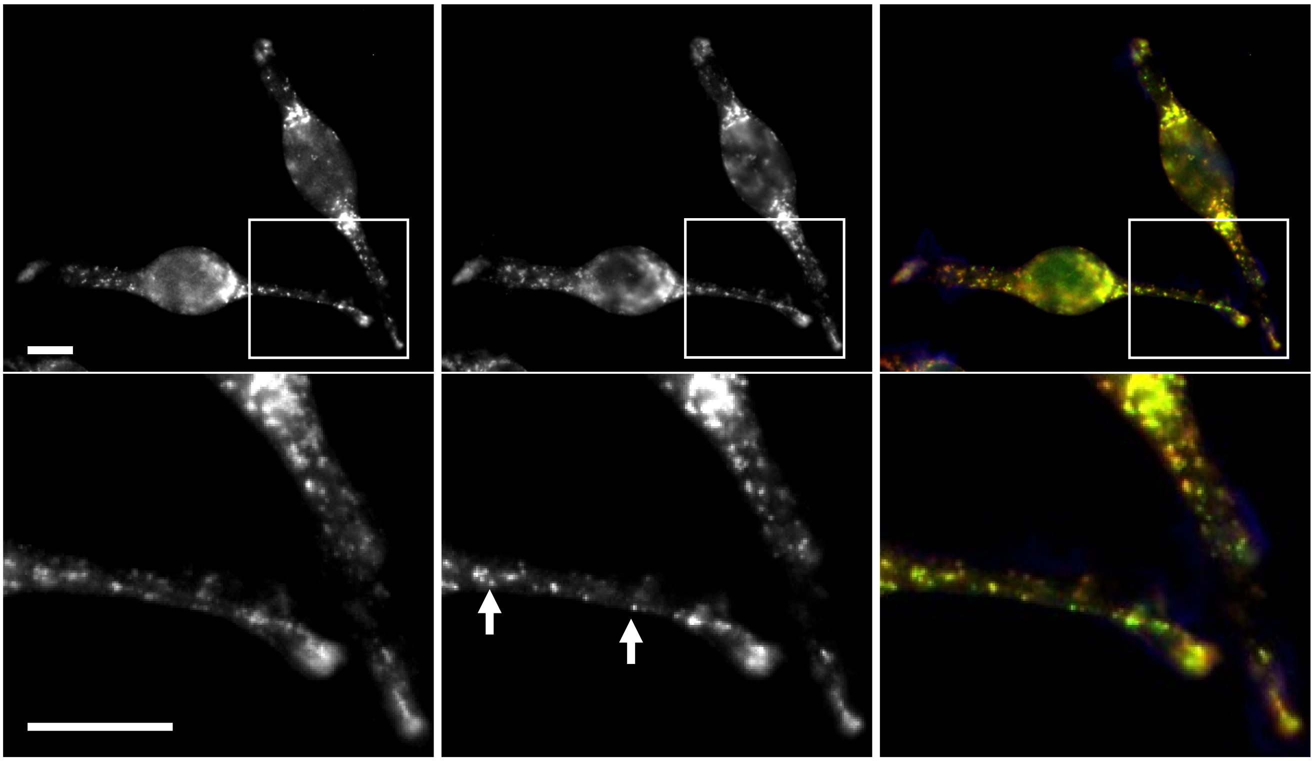

LysoTracker Red | CD63 (Green) | Merged

B Stimulated RBL-2H3 cells

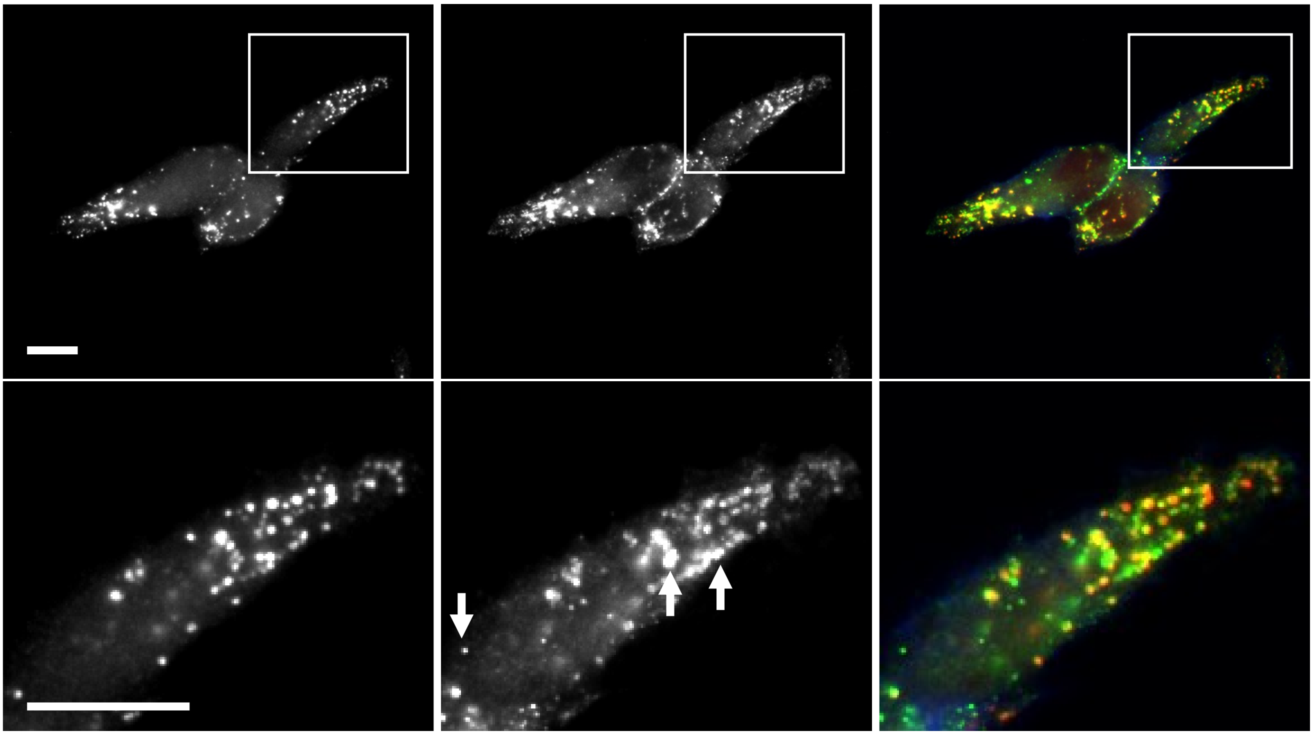

LysoTracker Red | CD63 (Green) | Merged

S2 Fig. LysoTracker labels CD63-positive vesicles in RBL-2H3 mast cells.
Live RBL-2H3 cells were labelled with LysoTracker Red (ThermoFisher). Cells were then left unstimulated (*A*), or antigen-stimulated for 20 min (*B*). Cells were fixed and CD63-positives vesicles were immuno-labelled with monoclonal CD63 antibodies (clone AD1, BioRad). F-actin was labelled with phalloidin iFluor-405 (Abcam). Images were taken with a Zeiss Observer Z1 epifluorescence microscope using a 63X 1.4 NA objective. Bottom panels show zoomed images of area indicated in the upper panels. Note that CD63 antibodies label many outlier vesicles that are not labelled with LysoTracker Red (*arrows*), while LysoTracker Red labelled vesicles predominately overlap with CD63 labelling. Scale bar, 10 µm.
