# Supplementary material for: Mast cell granule motility and exocytosis is driven by dynamic microtubule formation and kinesin-1 motor function
Source: PLoS One. 2022 Mar 22;17(3):e0265122. doi: 10.1371/journal.pone.0265122 (PMC8939832; doi:10.1371/journal.pone.0265122)
Supplement: S3 Fig — Colocalization of LysoTracker and CD63 labels in RBL-2H3 cells, as indicated in Figure Legend S3. Cells were imaged with a Leica UltraVIEW VoX spinning-disk confocal microscopy using a 63X, 1.4 NA objective. Colocalization of LysoTracker Red and CD63 signals was determined by calculation of Pearson’s correlation coefficient using the Volocity 6.1 plugin software. Seven images with at least 2 cells per image were analyzed; shown are representative analyses of unstimulated cells (A) and stimulated cells (B). Average Pearson’s correlation coefficients (r) were 0.714 +/-0.033 for unstimulated cells and 0.622 +/-0.055 for stimulated cells. Coefficients above 0.7 are generally considered strong correlation. Correlation is reduced during stimulation due to exocytosis which reduces LysoTracker labelling but not CD63. (PDF) [file pone.0265122.s003.pdf]

S3 Fig. Supporting Information

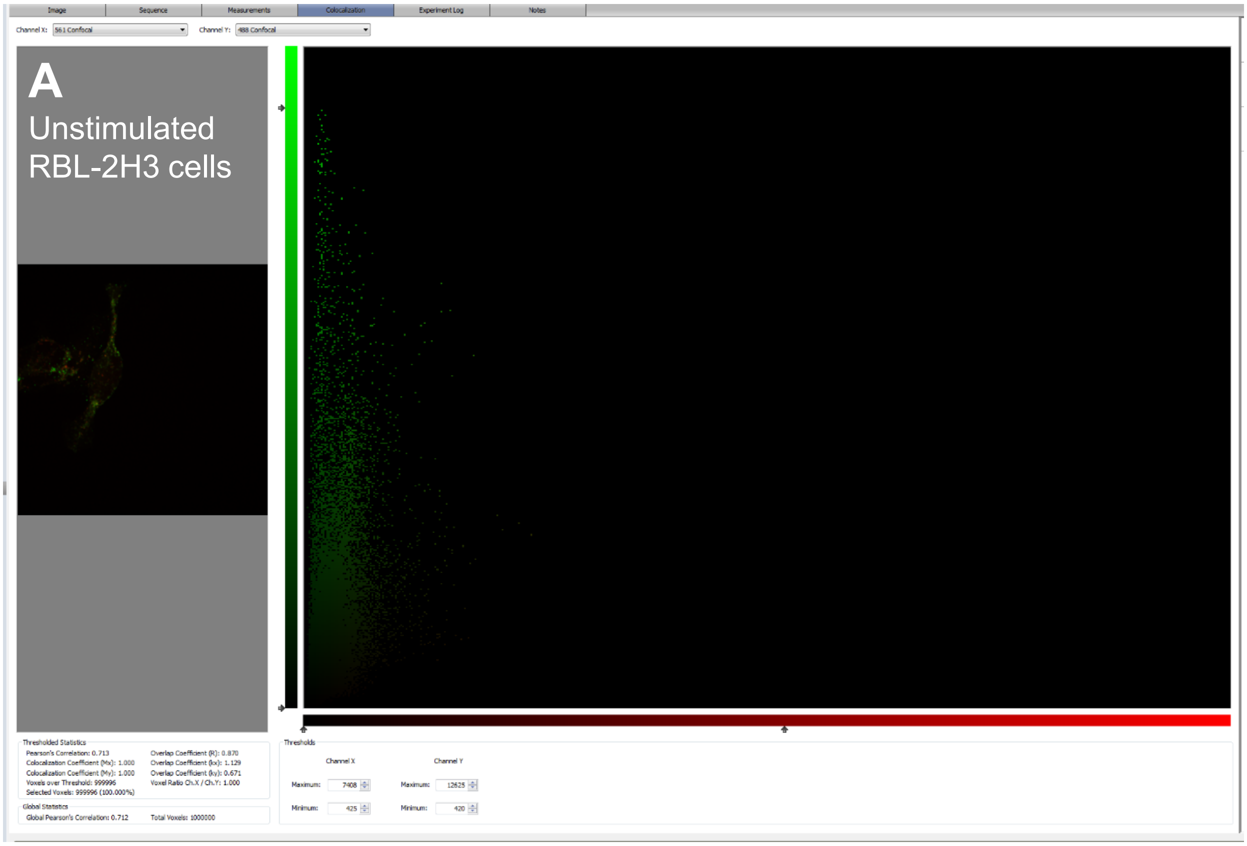

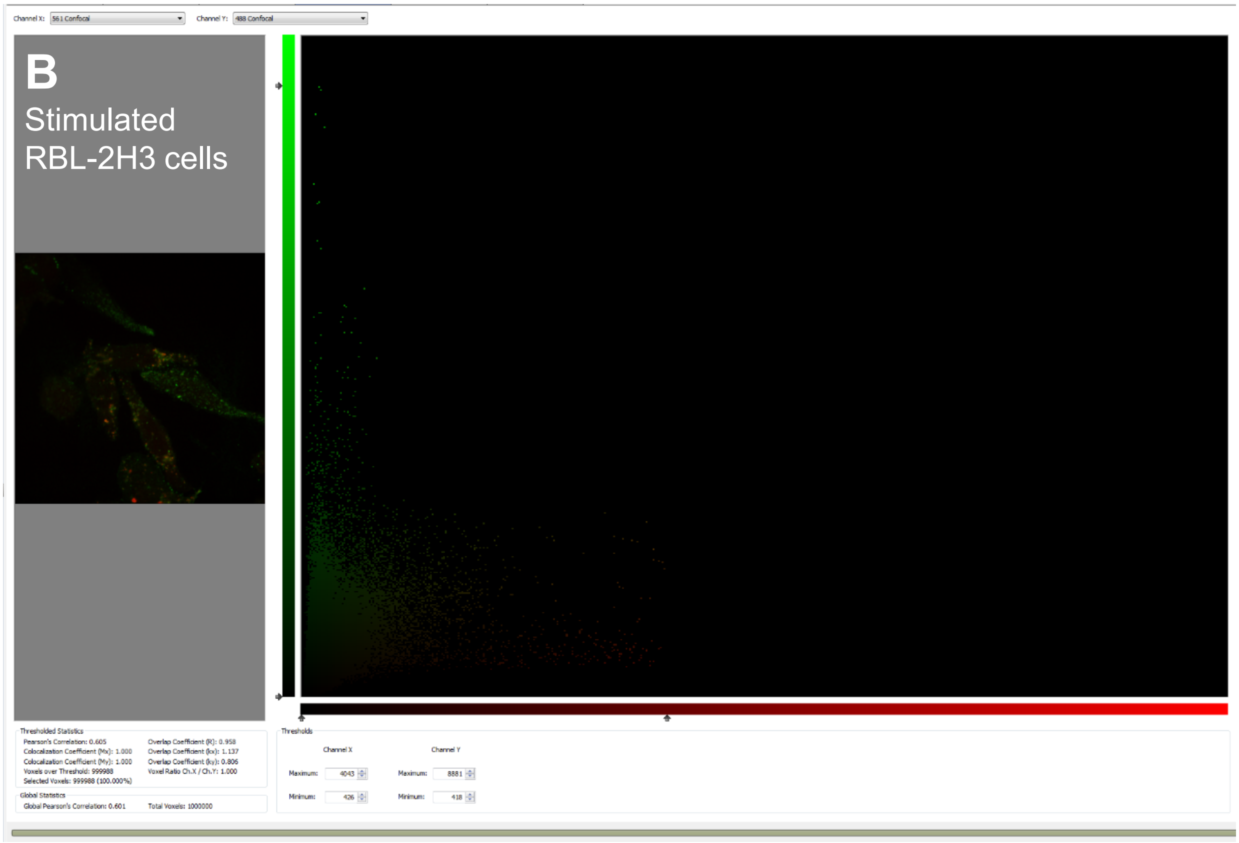

S3 Fig. Analysis of colocalization of LysoTracker and CD63 labels. Colocalization of LysoTracker and CD63 labels in RBL-2H3 cells, as indicated in Figure Legend S3. Cells were imaged with a Leica UltraVIEW VoX spinning-disk confocal microscopy using a 63X, 1.4 NA objective. Colocalization of LysoTracker Red and CD63 signals was determined by calculation of Pearson's correlation coefficient using the Volocity 6.1 plugin software. Seven images with at least 2 cells per image were analyzed; shown are representative analyses of unstimulated cells (*A*) and stimulated cells (*B*). Average Pearson's correlation coefficients (*r*) were 0.714 +/-0.033 for unstimulated cells and 0.622 +/-0.055 for stimulated cells. Coefficients above 0.7 are generally considered strong correlation. Correlation is reduced during stimulation due to exocytosis which reduces LysoTracker labelling but not CD63.
