# Supplementary material for: Mast cell granule motility and exocytosis is driven by dynamic microtubule formation and kinesin-1 motor function
Source: PLoS One. 2022 Mar 22;17(3):e0265122. doi: 10.1371/journal.pone.0265122 (PMC8939832; doi:10.1371/journal.pone.0265122)
Supplement: S1 Raw images — See the legend for Figs 5A and 7B and 7C for details. (PDF) [file pone.0265122.s004.pdf]

S1 Raw Images. Supporting Information

Fig 5A

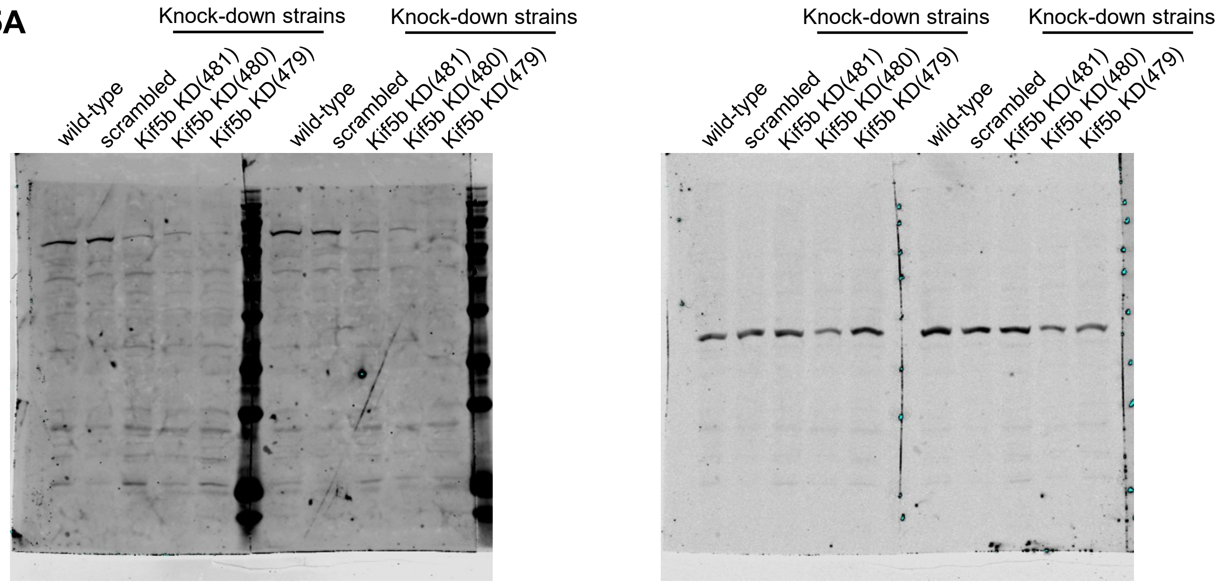

anti-Kif5b

anti-β-tubulin

Fig 7B

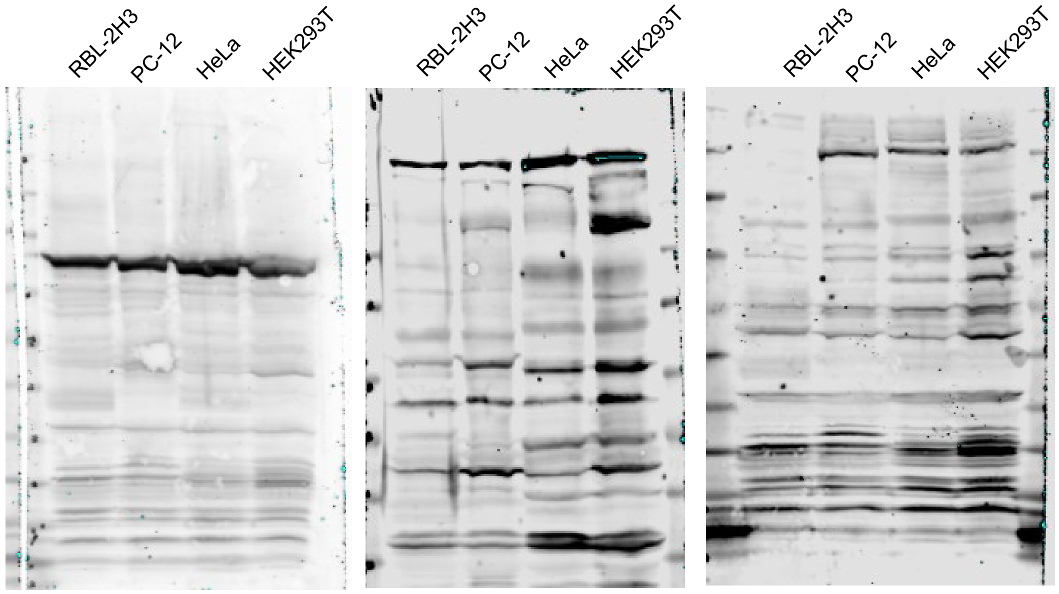

anti-Kif5b

anti-Slp3

anti-SKIP

Fig 7C

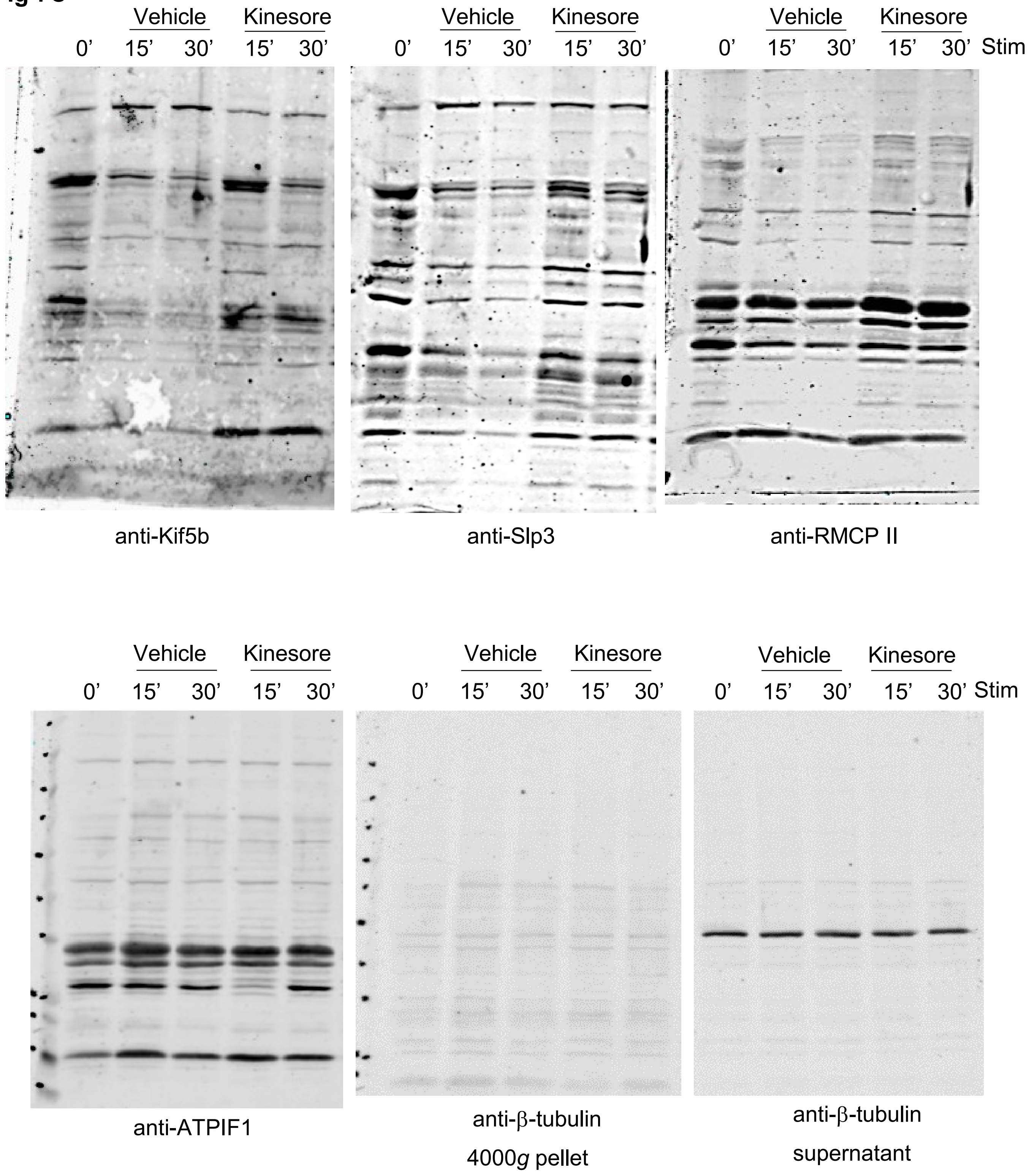

S1 Raw Images. Unaltered images for blots presented in Fig 5 and 7.
See the legend for Fig 5A and Fig 7B and 7C for details.
