# Supplementary material for: Mast cell granule motility and exocytosis is driven by dynamic microtubule formation and kinesin-1 motor function
Source: PLoS One. 2022 Mar 22;17(3):e0265122. doi: 10.1371/journal.pone.0265122 (PMC8939832; doi:10.1371/journal.pone.0265122)
Supplement: S1 File — See the figure legend for Figs 2A, 2E, 3A, 5B and 7A and 7D for details. (PDF) [file pone.0265122.s005.pdf]

S1 Raw Data. Supporting Information

S1 Raw Data. Raw data for results presented in Figures and Table 2. See the figure legend for Figs 2A, 2E, 3A, 5B, 7A and 7D for details.

Table 2 Raw Data

Sample	CD63 signal in perinuclear region (% of total)	s.e.m. (+/-)	*t*-test vs control / scrambled	
control (0.5% DMSO)	41.8	4.56	na	4a'
nocodazole (1 μM)	57.2	4.56	0.0219	4b'
colchicine (10 μM)	56.2	6.21	0.0662	4c'
paclitaxel (10 μM)	68.3	5.00	0.0006	4d'
kinesore (100 μM)	66.1	4.50	0.0009	4e'
scrambled (shRNA)	47.9	3.60	na	5c
Kif5b KD (shRNA)	59.7	3.69	0.0272	5d

Table 2 data

cell	Fig 4a' ROIpn	Fig 4a' ROIwc	Fig 4a' ratio (%)	Fig 4b' ROIpn	Fig 4b' ROIwc	Fig 4b' ratio (%)	Fig 4c' ROIpn	Fig 4c' ROIwc	Fig 4c' ratio (%)	Fig 4d' ROIpn	Fig 4d' ROIwc	Fig 4d' ratio (%)	Fig 4e' ROIpn	Fig 4e' ROIwc	Fig 4e' ratio (%)	Fig 5c ROIpn	Fig 5c ROIwc	Fig 5c ratio (%)	Fig 5d ROIpn	Fig 5d ROIwc	Fig 5d ratio (%)
1	528412.4	1054416.8	50.11419	254462	438621.8	58.01405	502435.2	877200.2	57.27714	385378.7	623265.6	61.83218	815451.9	1274755	63.96931	72535.2	102423.8	70.81872	195241.2	341141.84	57.23169
2	454119.8	1006422.56	45.12218	403566	682138.5	59.16181	548984.2	862865	63.62341	728752.9	1112729	65.49239	774357.7	1101055	70.32872	74283.92	137826.6	53.89663	254664.9	363811.2	69.99919
3	259136.6	792045.24	32.7174	317275	571617.5	55.50482	493778.5	845349.3	58.41118	440982.4	739619.9	59.62285	884418.4	1264043	69.96743	60009.96	103014.5	58.25391	148242.8	212549.89	69.74494
4	289475	984420.765	29.40562	445037	679723.3	65.47326	480640.2	862388.6	55.73359	655140.4	1083925	60.44148	580718.4	1001545	57.98223	64282.08	165764.7	38.77911	89634.27	222353.82	40.31155
5	403288.9	1219297.15	33.07552	238710	465481.4	51.28231	470528.7	818015	57.52079	372716.5	408391.2	91.26458	635953.6	1086853	58.51333	93763.72	184081.7	50.93592	187526.9	321554.54	58.31885
6	221130	914466.08	24.18133	460004	889608.4	51.70859	192010.3	935317.7	20.52889	269002.6	340825.8	78.92671	377105.1	605476.7	62.28235	94368.76	263941.2	35.75371	159912	242369.54	65.97859
7	530878	751781.005	70.61604	456605	738051.8	61.86631	328610.3	789174.5	41.63975	315355.1	353833	89.12541	336691.5	420230.9	80.12061	207204.8	481887.4	42.99859	352178.5	528683.38	66.61426
8	552433.3	1122916.94	49.19627	184852	213147.3	86.72507	271318	640948.8	42.33068	316148.1	479852.6	65.88442	492826.4	548377.1	89.86998	122349.6	277500.4	44.08989	174636.7	406911.46	42.91762
9	331503.6	678550.57	48.85466	152207	311365.3	48.88376	398916.5	491826	81.10927	532238.2	759364.2	70.08998	275958.4	696079.2	39.64468	166714.5	437128.5	38.13855	420440.5	595852.22	70.56121
10	264142.8	756454.06	34.91855	39723.9	119971	33.11125	359259.2	429019.7	83.73954	271620.1	665913.6	40.78909	395938.9	580295	68.23062	201883.1	444733.9	45.39413	166101.3	302600.64	54.89127
avg	383452	928077.117	41.82018	295244	510972.6	57.17312	404648.1	755210.5	56.19143	428733.5	656772	68.34691	556942	857870.9	66.09093	115739.6	259830.3	47.90592	214857.9	353782.853	59.65692
sd	126604.3	179361.2795	13.68291	145540	246105.6	13.67858	115142.9	173988.3	18.61542	160859.2	277596.7	15.00603	215505.1	320628.6	13.50482	56477.35	146856.5	10.79339	100537.5	127175.4408	11.0596
sem	44761.37	63413.7885	4.560971	51456	87011.47	4.559526	40709.16	61514.14	6.205139	56872.33	98145.25	5.00201	76192.56	113359.3	4.501606	19967.76	51921.62	3.597798	35545.37	44963.30828	3.686535
ttest vs 4a or 5c			na			0.021877			0.066193			0.000638			0.000855			na			0.027175

Table 2 Image J measurements

**ROIpn**	Area	mean	min	max	intDen	backgrnd	ROIpn (background c	**ROIwc**	Area	mean	min	max	intDen	backgrnd	ROIpn (background corrected)
4a' cell								4a' cell							
1	3734	150.353	5	231	561421	8.84	528412.4	1	11505	100.488	15	231	1156121	8.84	1054417
2	4405	111.932	7	227	493060	8.84	454119.8	2	23641	51.41	11	234	1215409	8.84	1006423
3	3729	78.332	5	215	292101	8.84	259136.6	3	15039	61.506	20	215	924990	8.84	792045.2
4	3889	79.48	0	228	309095	5.05	289475	4	12583	83.279	12	228	1047902	5.05	984420.8
5	3447	122.042	4	229	420679	5.05	403288.9	5	22730	58.688	4	232	1333970	5.05	1219297
6	3288	72.298	9	159	237718	5.05	221130	6	11976	81.403	13	186	974885	5.05	914466.1
7	3511	156.25	2	204	548591	5.05	530878	7	11511	70.355	2	204	809854	5.05	751781
8	4130	138.25	19	240	570977	4.49	552433.3	8	21994	55.545	6	240	1221670	4.49	1122917
9	3405	101.848	15	203	346792	4.49	331503.6	9	16407	45.847	1	203	752218	4.49	678550.6
10	4729	60.345	11	188	285376	4.49	264142.8	10	10206	78.608	19	188	802279	4.49	756454.1
4b' cell								4b' cell							
1	4949	55.167	7	231	273021	3.75	254462.3	1	13451	36.36	0	231	489063	3.75	438621.8
2	4722	89.215	24	224	421273	3.75	403565.5	2	12862	56.785	6	240	730371	3.75	682138.5
3	5289	63.738	7	239	337109	3.75	317275.3	3	19282	33.395	0	239	643925	3.75	571617.5
4	4612	100.245	19	238	462332	3.75	445037	4	13245	55.07	4	238	729392	3.75	679723.3
5	4582	53.297	29	254	244208	1.2	238709.6	5	12963	37.108	0	254	481037	1.2	465481.4
6	4215	110.335	28	255	465062	1.2	460004	6	15628	58.124	4	255	908362	1.2	889608.4
7	5938	78.095	9	241	463731	1.2	456605.4	7	17186	44.145	0	241	758675	1.2	738051.8

8	4107	47.559	3	188	195325	2.55	184852.2
9	4209	38.712	3	184	162940	2.55	152207.1
10	3742	13.166	3	172	49266	2.55	39723.9
4c' cell							
1	4265	121.924	21	192	520007	4.12	502435.2
2	3882	145.538	19	188	564978	4.12	548984.2
3	3954	129.001	24	204	510069	4.12	493778.5
4	4057	122.59	18	201	497355	4.12	480640.2
5	3761	129.227	11	196	486024	4.12	470528.7
6	3212	60.669	10	178	194869	0.89	192010.3
7	4930	67.545	10	141	332998	0.89	328610.3
8	4218	65.214	21	187	275072	0.89	271318
9	3507	115.818	20	240	406176	2.07	398916.5
10	3412	107.362	29	247	366322	2.07	359259.2
4d' cell							
1	4801	82.591	14	225	396517	2.32	385378.7
2	4644	159.243	7	221	739527	2.32	728752.9
3	5183	87.402	16	221	453007	2.32	440982.4
4	3802	174.634	9	209	663961	2.32	655140.4
5	4575	82.528	2	255	377566	1.06	372716.5
6	3957	69.04	3	251	273197	1.06	269002.6
7	4032	79.273	2	253	319629	1.06	315355.1
8	4331	74.056	6	232	320739	1.06	316148.1
9	3506	153.28	0	203	537392	1.47	532238.2
10	4121	67.38	0	196	277678	1.47	271620.1
4e' cell							
1	5118	160	14	229	818881	0.67	815451.9
2	4796	162.13	25	246	777571	0.67	774357.7
3	5189	171.1	17	254	887895	0.67	884418.4
4	4092	142.585	15	255	583460	0.67	580718.4
5	5126	124.734	25	254	639388	0.67	635953.6
6	4804	79.968	6	250	384167	1.47	377105.1
7	4484	76.557	7	207	343283	1.47	336691.5
8	4031	123.729	8	224	498752	1.47	492826.4
9	3997	70.511	6	207	281834	1.47	275958.4
10	3845	104.445	0	198	401591	1.47	395938.9
5c cell							
1	1990	39.97	0	250	79540	3.52	72535.2
2	2004	40.588	0	248	81338	3.52	74283.92
3	1402	46.323	0	255	64945	3.52	60009.96
4	1921	36.983	0	254	71044	3.52	64282.08
5	1354	70.069	0	191	94874	0.82	93763.72
6	1632	58.644	0	179	95707	0.82	94368.76
7	1853	116.731	11	227	216303	4.91	207204.8
8	2118	62.677	12	228	132749	4.91	122349.6
9	1994	88.5187	26	236	176505	4.91	166714.5
10	1701	123.595	14	204	210235	4.91	201883.1
5d cell							
1	1833	107.235	0	158	196561	0.72	195241.2
2	2696	95.18	0	182	256606	0.72	254664.9
3	1351	116.438	2	237	157308	6.71	148242.8
4	1663	60.609	0	253	100793	6.71	89634.27
5	1710	116.375	0	250	199001	6.71	187526.9
6	1400	120.933	0	255	169306	6.71	159912
7	2118	168.139	14	215	356118	1.86	352178.5
8	2055	86.841	6	241	178459	1.86	174636.7
9	2811	151.43	9	203	425669	1.86	420440.5
10	1388	121.53	3	224	168683	1.86	166101.3

8	12874	19.106	3	191	245976	2.55	213147.3
9	13245	26.058	0	184	345140	2.55	311365.3
10	10940	13.516	0	188	147868	2.55	119971
4c' cell							
1	13248	70.334	0	192	931782	4.12	877200.2
2	12875	71.138	0	197	915910	4.12	862865
3	13464	66.906	2	204	900821	4.12	845349.3
4	12745	71.785	6	201	914898	4.12	862388.6
5	15117	58.232	0	196	880297	4.12	818015
6	10097	93.523	0	179	944304	0.89	935317.7
7	12977	61.703	0	141	800724	0.89	789174.5
8	11207	58.082	0	187	650923	0.89	640948.8
9	21514	24.93	0	244	536360	2.07	491826
10	20804	22.692	0	247	472084	2.07	429019.7
4d' cell							
1	18895	35.306	0	227	667102	2.32	623265.6
2	20231	57.321	0	221	1159665	2.32	1112729
3	20819	37.846	0	230	787920	2.32	739619.9
4	20231	55.897	0	227	1130861	2.32	1083925
5	13180	32.046	0	255	422362	1.06	408391.2
6	11620	30.39	0	251	353143	1.06	340825.8
7	10884	33.57	0	253	365370	1.06	353833
8	11506	42.765	4	232	492049	1.06	479852.6
9	11506	67.467	0	206	776278	1.47	759364.2
10	11620	58.778	0	211	682995	1.47	665913.6
4e' cell							
1	23839	54.144	0	229	1290727	0.67	1274755
2	23299	47.928	0	246	1116665	0.67	1101055
3	23230	55.084	0	254	1279607	0.67	1264043
4	21401	47.469	9	255	1015884	0.67	1001545
5	23647	46.63	0	254	1102696	0.67	1086853
6	25224	25.474	0	250	642556	1.47	605476.7
7	21545	20.975	0	207	451902	1.47	420230.9
8	19004	30.326	9	224	576313	1.47	548377.1
9	20474	35.468	1	207	726176	1.47	696079.2
10	21832	28.05	0	198	612388	1.47	580295
5c cell							
1	7937	16.425	0	250	130362	3.52	102423.8
2	9243	18.431	0	248	170362	3.52	137826.6
3	5426	22.5	0	255	122114	3.52	103014.5
4	9864	20.325	0	254	200486	3.52	165764.7
5	6793	27.908	0	191	189652	0.82	184081.7
6	8412	32.197	0	179	270839	0.82	263941.2
7	6804	75.734	0	227	515295	4.91	481887.4
8	7493	41.945	0	228	314291	4.91	277500.4
9	6983	67.509	0	236	471415	4.91	437128.5
10	9243	53.026	0	204	490117	4.91	444733.9
5d cell							
1	8303	41.807	0	158	347120	0.72	341141.8
2	9490	39.056	0	182	370644	0.72	363811.2
3	5241	47.265	0	237	247717	6.71	212549.9
4	6158	42.818	0	253	263674	6.71	222353.8
5	4626	76.22	0	250	352595	6.71	321554.5
6	5926	47.61	0	255	282133	6.71	242369.5
7	11467	47.965	0	215	550012	1.86	528683.4
8	13839	31.263	0	241	432652	1.86	406911.5
9	9423	65.092	0	203	613379	1.86	595852.2
10	8926	35.761	0	224	319203	1.86	302600.6

Fig 2A Raw Data: Effect of microtubule drugs on exocytosis

uM drug	colchicine Exocytos (normal)	s.e.m.
0	100	0
0.001	85.01	3.25
0.01	93.57	7.16
0.1	74.75	10.20
1	64.30	10.30
10	43.00	5.37

uM drug	paclitaxel Exocytos (normal)	s.e.m.
0	100	0
0.001	93.66	3.81
0.01	87.69	5.96
0.1	75.27	7.09
1	69.09	5.09
10	64.23	5.18

uM drug	nocodazole Exocytos (normal)	s.e.m.
0	100	0
0.001	92.10656	5.18357
0.01	72.78303	11.18896
0.1	34.1439	9.554179
1	18.24428	7.691461
10	12.82846	5.274907

Trial1

Colchicine	0	0.001	0.01	0.1	1	10	
Rest	2014	2502	2501	1807	1862	1731	360,460
Stim1	33057	30777	29620	29234	25045	13823	360,460
Stim2	32345	30951	26708	26748	24193	14660	360,460
Stim3	29806	29394	28881	25057	23737	15074	360,460
Lyse R	45199	47344	45190	43066	43952	43415	360,460
Lyse S1	22621	24462	23895	24035	24354	33931	360,460
Lyse S2	22208	26224	25725	27209	27368	30811	360,460
Lyse S3	23533	28047	28329	27417	28907	36051	360,460

Trial2

Colchicine	0	0.001	0.01	0.1	1	10	
Rest	5974	7871	4560	5720	7058	6385	360,460
Stim1	30652	26457	23040	22511	17946	14091	360,460
Stim2	26577	21157	22394	22710	17280	15709	360,460
Stim3	23329	21736	25468	15804	14449	16463	360,460
Lyse R	45891	42785	45685	39470	39276	39795	360,460
Lyse S1	24151	20828	20422	22148	21949	31359	360,460
Lyse S2	23505	24403	21583	25420	23111	32444	360,460
Lyse S3	24151	26481	22263	25903	31652	31671	360,460

Trial3

Colchicine	0	0.001	0.01	0.1	1	10	
Rest	3711	3349	3463	5433	5865	5276	360,460
Stim1	22591	14724	13966	11456	11762	11628	360,460
Stim2	20199	13256	13141	12109	10652	10450	360,460
Stim3	22062	15538	15366	12128	10968	9782	360,460
Lyse R	46559	33831	36593	32261	42052	37380	360,460
Lyse S1	42249	28185	21774	23904	26867	31311	360,460
Lyse S2	30669	27452	23165	23551	26105	38876	360,460
Lyse S3	29087	23691	28566	24662	28555	35199	360,460

Exo	0	0.001	0.01	0.1	1	10
Rest	4.27	5.02	5.24	4.03	4.06	3.83
Stim1	59.37	55.72	55.35	54.88	50.70	28.95
Stim2	59.29	54.13	50.94	49.57	46.92	32.24
Stim3	55.88	51.17	50.48	47.75	45.09	29.48

Exo	0	0.001	0.01	0.1	1	10
Rest	11.52	15.54	9.08	12.66	15.23	13.83
Stim1	55.93	55.95	53.01	50.41	44.98	31.00
Stim2	53.07	46.44	50.92	47.18	42.78	32.62
Stim3	49.13	45.08	53.36	37.89	31.34	34.20

Exo	0	0.001	0.01	0.1	1	10
Rest	7.38	9.01	8.65	14.41	12.24	12.37
Stim1	34.84	34.31	39.08	32.40	30.45	27.08
Stim2	39.71	32.56	36.20	33.96	28.98	21.19
Stim3	43.13	39.61	34.98	32.97	27.75	21.75

avg - rest	Exo col1	53.92	48.65	47.01	46.71	43.51	26.39
	Exo col2	41.19	33.62	43.35	32.50	24.47	18.78
	Exo col3	31.85	26.49	28.10	18.69	16.82	10.97
normalized	Exo col1	100	90.24289	87.1963	86.63183	80.69297	48.94629
	Exo col2	100	81.61275	105.2495	78.90679	59.40266	45.59879

Trial1

Taxol	0	0.001	0.01	0.1	1	10	
Rest	3444	2547	3194	2836	3547	3376	360,460
Stim1	13830	11662	11043	9612	9068	9674	360,460
Stim2	13311	12055	12047	10280	11006	10032	360,460
Stim3	13540	12545	11453	10224	10239	9965	360,460
Lyse R	49389	47737	47868	49290	48871	49140	360,460
Lyse S1	36070	38910	39422	41344	41714	41392	360,460
Lyse S2	37939	38493	37527	43086	40684	44298	360,460
Lyse S3	38830	40838	43058	43616	47730	48555	360,460

Trial2

Taxol	0	0.001	0.01	0.1	1	10	
Rest	2050	3278	2095	4034	2586	3022	360,460
Stim1	12033	12377	12307	12347	12657	11267	360,460
Stim2	12044	16703	11621	12654	10818	10057	360,460
Stim3	13165	15446	10981	11809	9605	10229	360,460
Lyse R	42894	40917	46926	40404	44720	49263	360,460
Lyse S1	31836	35336	34221	35523	41074	35868	360,460
Lyse S2	36478	36697	34235	39688	39630	39645	360,460
Lyse S3	38661	38198	36523	40099	41305	41985	360,460

Trial3

Taxol	0	0.001	0.01	0.1	1	10	
Rest	2151	2708	2453	2497	2679	3272	360,460
Stim1	11739	12085	11145	12338	10296	9420	360,460
Stim2	13232	11787	10581	9690	9123	6270	360,460
Stim3	12558	11060	11390	10973	9192	8643	360,460
Lyse R	30554	45839	45630	46586	45963	48456	360,460
Lyse S1	27185	33346	33502	34093	35885	27471	360,460
Lyse S2	29557	33935	34345	35066	30942	34086	360,460
Lyse S3	37362	35555	37048	33443	38002	37185	360,460

Exo	0	0.001	0.01	0.1	1	10
Rest	6.52	5.07	6.26	5.44	6.77	6.43
Stim1	27.72	23.06	21.88	18.86	17.86	18.94
Stim2	25.97	23.85	24.30	19.26	21.29	18.46
Stim3	25.85	23.50	21.01	18.99	17.66	17.03

Exo	0	0.001	0.01	0.1	1	10
Rest	4.56	7.42	4.27	9.08	5.47	5.78
Stim1	27.43	25.94	26.45	25.79	23.56	23.90
Stim2	24.82	31.28	25.34	24.18	21.44	20.23
Stim3	25.40	28.79	23.12	22.75	18.87	19.59

Exo	0	0.001	0.01	0.1	1	10
Rest	6.58	5.58	5.10	5.09	5.51	6.33
Stim1	30.16	26.60	24.96	26.57	22.29	25.53
Stim2	30.92	25.78	23.55	21.65	22.77	15.54
Stim3	25.16	23.73	23.51	24.71	19.48	18.86

avg - res	Exo tax1	20.00	18.40	16.14	13.60	12.17	11.72
	Exo tax2	21.32	21.25	20.70	15.16	15.82	15.46
	Exo tax3	22.17	19.79	18.91	19.22	16.01	13.65
normaliz	Exo tax1	100	92.04233	80.73222	68.00537	60.86629	58.59948
	Exo tax2	100	99.67477	97.05844	71.10363	74.20245	72.51746

Trial1

Noco	0	0.001	0.01	0.1	1	10	
Rest	18103	17513	16207	17870	14401	14520	360,460
Stim1	71757	73194	44610	27063	15616	17972	360,460
Stim2	80450	70532	50519	29516	14858	15329	360,460
Stim3	131628	80859	49695	20938	16307	16069	360,460
Lyse R	203421	199145	184027	212130	196218	199973	360,460
Lyse S1	147235	142278	143238	149942	147518	177735	360,460
Lyse S2	145728	145724	138217	145264	149404	174530	360,460
Lyse S3	136390	140208	130258	150204	144236	169406	360,460

Trial2

Noco	0	0.001	0.01	0.1	1	10	
Rest	26526	29892	31786	31715	24359	21058	360,460
Stim1	128272	123643	123928	54383	34283	24693	360,460
Stim2	121453	112763	110571	57533	35251	25353	360,460
Stim3	129463	115608	114489	60773	44296	25781	360,460
Lyse R	238479	227653	224852	226712	226526	231733	360,460
Lyse S1	144529	148016	146819	130876	137883	130876	360,460
Lyse S2	146842	137874	143912	130480	150991	128782	360,460
Lyse S3	156423	152725	145021	144436	150452	145476	360,460

Trial3

Noco	0	0.001	0.01	0.1	1	10	
Rest	30219	31517	31726	30361	30026	31517	360,460
Stim1	87739	99647	95998	40835	30928	35322	360,460
Stim2	101221	106391	95315	33133	31938	30389	360,460
Stim3	70386	94500	105933	35575	29239	32027	360,460
Lyse R	208804	239444	119481	227549	225671	220067	360,460
Lyse S1	145675	173074	153891	152123	175363	176876	360,460
Lyse S2	152564	162465	156536	147643	160204	179707	360,460
Lyse S3	126173	184974	157825	152282	160439	178160	360,460

Exo	0	0.001	0.01	0.1	1	10
Rest	8.17	8.08	8.09	7.77	6.84	6.77
Stim1	32.77	33.97	23.75	15.29	9.57	9.18
Stim2	35.57	32.62	26.77	16.89	9.05	8.07
Stim3	49.11	36.58	27.62	12.23	10.16	8.66

Exo	0	0.001	0.01	0.1	1	10
Rest	10.01	11.61	12.39	12.27	9.71	8.33
Stim1	47.02	45.51	45.77	29.36	19.91	15.87
Stim2	45.27	44.99	43.45	30.60	18.93	16.45
Stim3	45.28	43.08	44.12	29.62	22.75	15.05

Exo	0	0.001	0.01	0.1	1	10
Rest	12.64	11.63	20.98	11.77	11.74	12.53
Stim1	37.59	36.54	38.42	21.16	14.99	16.65
Stim2	39.88	39.57	37.85	18.33	16.62	14.46
Stim3	35.81	33.81	40.16	18.94	15.42	15.24

avg - res	Exo noc1	30.98	26.30	17.95	7.03	2.75	1.87
	Exo noc2	35.85	32.92	32.06	17.58	10.82	7.46
	Exo noc3	25.12	25.01	17.83	7.70	3.93	2.92
normaliz	Exo noc1	100	84.91294	57.94398	22.70751	8.891263	6.03921
	Exo noc2	100	91.83974	89.43498	49.05293	30.18081	20.81426

**Exo col3**	100	83.17681	88.25176	58.70045	52.81697	34.44418
**Exo col avg**	100	85.01082	93.56584	74.74636	64.3042	42.99642
**Exo col sem**	0	3.251349	7.164461	10.19859	10.30259	5.369216

**Exo tax3**	100	89.27125	85.28962	86.70643	72.20143	61.57893
**Exo tax avg**	100	93.66279	87.69343	75.27181	69.09006	64.23196
**Exo tax sem**	0	3.809705	5.956934	7.087408	5.085458	5.182012

**Exo noc3**	100	99.56701	70.97014	30.67127	15.66076	11.63191
**Exo noc avg**	100	92.10656	72.78303	34.1439	18.24428	12.82846
**Exo noc sem**	0	5.18357	11.18896	9.554179	7.691461	5.274907

Data for Fig 2A

	colchicine	
uM drug	tosis (norm	s.e.m.
0	100.00	0.00
0.001	85.01	3.25
0.01	93.57	7.16
0.1	74.75	10.20
1	64.30	10.30
10	43.00	5.37

	paclitaxel	
uM drug	tosis (norm	s.e.m.
0	100.00	0.00
0.001	93.66	3.81
0.01	87.69	5.96
0.1	75.27	7.09
1	69.09	5.09
10	64.23	5.18

	nocodazole	
uM drug	tosis (norm	s.e.m.
0	100.00	0.00
0.001	92.11	5.18
0.01	72.78	11.19
0.1	34.14	9.55
1	18.24	7.69
10	12.83	5.27

Fig 2A Data: Granule speed

particle tracking for lysotracker labelled cells

control	0.456556	nocodazo	0.494408	colchicine	0.382685	taxol	0.244484
sem	0.216769	sem	0.253053	sem	0.241878	sem	0.099559

granule speed EB3 DMSO control

Item Name	Populatior	Type	Track ID	Angle (deg	Bearing (d	Length (µn	Time Span	Track Velocity (µm/sec)
20190327_	Populatior	Track	45384	115.53	244.47	7.49	11	0.277
20190327_	Populatior	Track	45388	152.56	207.44	0.437	4	0.0726
20190327_	Populatior	Track	45392	109.49	109.49	3.19	4	0.53
20190327_	Populatior	Track	45401	15	15	9.15	9	0.436
20190327_	Populatior	Track	45405	150.54	150.54	2.03	4	0.339
20190327_	Populatior	Track	45408	35.36	324.64	0.413	3	0.138
20190327_	Populatior	Track	45457	24.2	24.2	4.7	4	0.784
20190327_	Populatior	Track	45461	169.56	190.44	2.96	4	0.493
20190327_	Populatior	Track	45464	11.01	348.99	3.19	3	1.06
20190327_	Populatior	Track	45467	131.63	228.37	1.12	3	0.372
20190327_	Populatior	Track	45479	174.63	185.37	13.28	12	0.443
20190327_	Populatior	Track	45482	0.35	0.35	1.42	3	0.475
20190327_	Populatior	Track	45485	71.05	71.05	0.848	3	0.282
20190327_	Populatior	Track	45488	87.59	272.41	0.688	3	0.229
20190327_	Populatior	Track	45491	130.07	229.93	1.8	3	0.6
20190327_	Populatior	Track	45494	34.58	34.58	2.85	3	0.951
20190327_	Populatior	Track	45500	51.89	51.89	1.83	6	0.153
20190327_	Populatior	Track	45503	86.25	86.25	0.667	3	0.222
20190327_	Populatior	Track	45519	14.75	14.75	12.54	16	0.299
20190327_	Populatior	Track	45536	57.3	57.3	4.83	17	0.107
20190327_	Populatior	Track	45539	81.14	278.86	1.57	3	0.523
20190327_	Populatior	Track	45543	144.2	144.2	1.04	4	0.174
20190327_	Populatior	Track	45546	81.7	81.7	1.08	3	0.359
20190327_	Populatior	Track	45551	166.3	166.3	5.9	5	0.656
20190327_	Populatior	Track	45557	28.81	331.19	5.6	6	0.467
20190327_	Populatior	Track	45560	43.26	316.74	2.18	3	0.727

20190327_Populatior Track	45564	35.83	35.83	2.08	4	0.346
20190327_Populatior Track	45576	121.55	121.55	17.05	12	0.568
20190327_Populatior Track	45588	55.52	304.48	5.47	12	0.182
20190327_Populatior Track	45591	34.13	34.13	1.56	3	0.522
20190327_Populatior Track	45597	174.49	174.49	4.86	6	0.405
20190327_Populatior Track	45600	149.78	210.22	1.32	3	0.441
20190327_Populatior Track	45603	168.73	191.27	0.239	3	0.0797
20190327_Populatior Track	45609	163.81	163.81	0.716	3	0.238
20190327_Populatior Track	45615	106.89	106.89	8.49	6	0.707
20190327_Populatior Track	45620	12.37	347.63	2.63	5	0.292
20190327_Populatior Track	45623	69.44	290.56	0.507	3	0.169
20190327_Populatior Track	45626	139.27	220.73	1.08	3	0.36
20190327_Populatior Track	45630	140.04	219.96	2.73	4	0.457
20190327_Populatior Track	45633	68.76	291.24	0.42	3	0.14
20190327_Populatior Track	45638	26.63	333.37	6.34	5	0.705
20190327_Populatior Track	45641	3.42	356.58	1.72	3	0.574
20190327_Populatior Track	45657	79.79	79.79	25.49	16	0.607
					avg sp	
20190327_Populatior Track	46705	14.39	345.61	7.59	8	0.421
20190327_Populatior Track	46711	6.09	353.91	0.371	3	0.124
20190327_Populatior Track	46771	32.72	32.72	15.91	33	0.171
20190327_Populatior Track	46786	25.22	334.78	4.93	8	0.274
20190327_Populatior Track	46795	126.33	233.67	6.64	5	0.738
20190327_Populatior Track	46833	146.12	213.88	25.87	21	0.454
20190327_Populatior Track	46841	112.45	247.55	2.28	4	0.38
20190327_Populatior Track	46846	125.67	234.33	0.815	3	0.272
20190327_Populatior Track	46852	145.12	214.88	1.36	3	0.454
20190327_Populatior Track	46863	172.48	187.52	8.58	6	0.715
20190327_Populatior Track	46872	124.65	124.65	3.76	5	0.418
20190327_Populatior Track	46885	160.14	199.86	13.64	7	0.91
20190327_Populatior Track	46892	32.46	32.46	3.08	4	0.514
20190327_Populatior Track	46905	133.1	226.9	3.14	7	0.209
20190327_Populatior Track	46917	164.76	195.24	1.98	4	0.33

20190327_Populatior Track	46923	154.63	205.37	3.03	3	0.0101
20190327_Populatior Track	46928	46.06	46.06	0.59	3	0.197
20190327_Populatior Track	46960	53.19	53.19	17.11	17	0.38
20190327_Populatior Track	46993	116.39	116.39	14.66	18	0.305
20190327_Populatior Track	46998	122.11	237.89	0.762	3	0.254
20190327_Populatior Track	47004	89.73	89.73	1.61	3	0.536
20190327_Populatior Track	47011	42.33	317.67	1.28	4	0.213
20190327_Populatior Track	47026	62.09	62.09	9.42	8	0.523
20190327_Populatior Track	47048	149.84	210.16	13.45	12	0.448
20190327_Populatior Track	47062	86.97	86.97	11.62	8	0.646
20190327_Populatior Track	47070	9.18	350.82	2.3	4	0.383
20190327_Populatior Track	47079	28.83	331.17	6.82	5	0.757
20190327_Populatior Track	47103	125.51	125.51	12.98	13	0.393
20190327_Populatior Track	47119	106.49	106.49	4.69	9	0.223
20190327_Populatior Track	47125	90.41	269.59	0.962	3	0.321
20190327_Populatior Track	47132	146.89	213.11	2.55	4	0.425
20190327_Populatior Track	47139	141.75	141.75	5.96	4	0.993
20190327_Populatior Track	47142	170.12	170.12	11.37	11	0.421
20190327_Populatior Track	47167	22.92	337.08	5.13	4	0.854
20190327_Populatior Track	47172	35.47	35.47	2.86	3	0.952
20190327_Populatior Track	47185	16.38	16.38	5.98	7	0.399
20190327_Populatior Track	47191	50.05	50.05	1.44	3	0.48
20190327_Populatior Track	47196	16.95	16.95	0.567	3	0.189
20190327_Populatior Track	47204	144.36	144.36	6.16	4	1.53
20190327_Populatior Track	47213	84.64	84.64	6.12	5	0.68
20190327_Populatior Track	47237	146.5	213.5	12.36	10	0.515
20190327_Populatior Track	47242	96.59	96.59	2.23	3	0.746
20190327_Populatior Track	47325	39.88	39.88	0.966	3	0.322
20190327_Populatior Track	47332	13.03	346.97	4.3	4	0.716
20190327_Populatior Track	47351	34.45	325.55	9.12	10	0.38
20190327_Populatior Track	47356	57.58	57.58	1.67	3	0.557
20190327_Populatior Track	47362	111.26	111.26	0.256	3	0.0854
20190327_Populatior Track	47367	10.31	10.31	0.278	3	0.0929
20190327_Populatior Track	47375	21.61	21.61	5.6	4	0.933

20190327_Populatior Track	47380	158.95	201.05	1.64	3	0.547
20190327_Populatior Track	47399	134.21	134.21	18.31	10	0.763
20190327_Populatior Track	47406	44.2	44.2	2.48	4	0.413
20190327_Populatior Track	47413	34.61	325.39	5.54	4	0.923
20190327_Populatior Track	47423	150.15	209.85	6.69	5	0.743
20190327_Populatior Track	47432	23.78	23.78	5.26	5	0.585
				avg sp		
20190327_Populatior Track	55805	134.68	225.32	1.75	3	0.585
20190327_Populatior Track	55810	34.29	34.29	0.543	3	0.181
20190327_Populatior Track	55855	129.69	230.31	19.49	25	0.283
20190327_Populatior Track	55864	71.18	71.18	7.38	5	0.821
20190327_Populatior Track	55880	52.68	52.68	14.57	9	0.696
20190327_Populatior Track	55888	162	198	5.12	5	0.569
20190327_Populatior Track	55896	86.67	273.33	2.53	4	0.422
20190327_Populatior Track	55901	110.48	249.52	0.285	3	0.0951
20190327_Populatior Track	55906	150	210	0.821	3	0.274
20190327_Populatior Track	55913	7.43	352.57	2.83	4	0.473
20190327_Populatior Track	55927	105.1	254.9	7.88	8	0.438
20190327_Populatior Track	55933	169.07	190.93	2.37	3	0.79
20190327_Populatior Track	55940	119.95	240.05	0.922	4	0.154
20190327_Populatior Track	55951	122.87	122.87	1.13	6	0.0946
20190327_Populatior Track	55963	14.9	345.1	7.22	7	0.481
20190327_Populatior Track	55968	74.92	74.92	2.53	3	0.0842
20190327_Populatior Track	55979	168.09	191.91	4.41	6	0.367
20190327_Populatior Track	55991	25.07	334.93	3.22	7	0.215
20190327_Populatior Track	56022	137.21	222.79	14.29	17	0.318
20190327_Populatior Track	56030	28.7	331.3	4.08	5	0.454
20190327_Populatior Track	56036	162.24	197.76	1.26	3	0.42
20190327_Populatior Track	56043	88.62	88.62	3.06	4	0.512
20190327_Populatior Track	56048	121.84	238.16	2.77	3	0.92
20190327_Populatior Track	56061	50.62	50.62	8.25	7	0.549
20190327_Populatior Track	56066	89.13	89.13	0.345	3	0.115
20190327_Populatior Track	56071	44.43	315.57	1.61	3	0.538

20190327_	Populatior Track	56089	124.71	235.29	9.37	10	0.39
20190327_	Populatior Track	56110	150.34	150.34	11.03	12	0.368
20190327_	Populatior Track	56116	24.6	24.6	0.0717	3	0.0239
20190327_	Populatior Track	56141	29.94	29.94	16.25	14	2.451
20190327_	Populatior Track	56178	84.91	275.09	20.31	21	0.356
20190327_	Populatior Track	56190	179.5	179.5	5.94	7	0.396
20190327_	Populatior Track	56205	168.01	168.01	2.52	8	0.14
20190327_	Populatior Track	56214	174.64	185.36	5	5	0.556
20190327_	Populatior Track	56223	45.7	314.3	2.16	5	0.24
20190327_	Populatior Track	56231	147.08	212.92	2.07	5	0.23
						avg sp	
					granule sp	control	0.456556
						sem	0.216769

granule speed EB3 nocodazole

Item Name	Populatior Type	Track ID	Angle (deg	Bearing (d	Length (µn	Time Span	Track Velocity (µm/sec)
20190322_	Populatior Track	103111	151.38	151.38	13.04	13	0.395
20190322_	Populatior Track	103119	30.32	30.32	17.92	8	0.996
20190322_	Populatior Track	103125	101.08	101.08	8.6	6	0.717
20190322_	Populatior Track	103129	45.66	314.34	11.05	4	1.84
20190322_	Populatior Track	103132	66.4	293.6	0.516	3	0.172
20190322_	Populatior Track	103141	46.04	313.96	24.55	9	1.17
20190322_	Populatior Track	103144	164	196	0.329	3	0.11
20190322_	Populatior Track	103148	74.45	285.55	1.16	4	0.193
20190322_	Populatior Track	103151	9.43	9.43	0.652	3	0.217
20190322_	Populatior Track	103165	138.63	221.37	42.71	14	1.19
20190322_	Populatior Track	103186	39.28	320.72	22.34	21	0.392
20190322_	Populatior Track	103206	129.66	129.66	8.88	20	0.165
20190322_	Populatior Track	103279	79.12	79.12	0.375	3	0.125
20190322_	Populatior Track	103282	169.65	190.35	0.235	3	0.0784
20190322_	Populatior Track	103287	66.06	66.06	2.47	5	0.274
20190322_	Populatior Track	103290	40.59	319.41	0.917	3	0.306
20190322_	Populatior Track	103303	165.52	194.48	11.15	13	0.337

20190322_Populatior Track	103309	51.5	308.5	16.98	6	1.14
20190322_Populatior Track	103316	142.17	217.83	3.53	7	0.235
20190322_Populatior Track	103334	89.4	270.6	24.13	18	0.503
20190322_Populatior Track	103338	176.76	176.76	3.91	4	0.651
20190322_Populatior Track	103345	171.36	171.36	7.9	7	0.526
20190322_Populatior Track	103351	139.78	220.22	2.94	6	0.245
20190322_Populatior Track	103364	56.42	303.58	5.52	13	0.167
20190322_Populatior Track	103373	166.58	166.58	4.93	9	0.235
20190322_Populatior Track	103397	91.75	91.75	11.23	24	0.17
20190322_Populatior Track	103406	109.31	109.31	27.22	9	1.3
20190322_Populatior Track	103409	0.171	359.83	1.46	3	0.487
20190322_Populatior Track	103444	162.72	162.72	48.89	35	0.494
20190322_Populatior Track	103450	11.74	348.26	16.79	6	1.4
20190322_Populatior Track	103453	90.84	90.84	0.121	3	0.0403
20190322_Populatior Track	103456	130.96	130.96	0.187	3	0.0624
20190322_Populatior Track	103468	144	216	4.92	12	0.164
20190322_Populatior Track	103477	11.26	348.74	12.95	9	0.617
20190322_Populatior Track	103485	119.6	240.4	2.77	8	0.154
20190322_Populatior Track	103489	32.86	327.14	1.03	4	0.172
20190322_Populatior Track	103494	32.17	32.17	3.44	5	0.382
20190322_Populatior Track	103507	125.63	125.63	8.26	13	0.25
20190322_Populatior Track	103513	88.36	271.64	10.44	6	0.87
20190322_Populatior Track	103519	99.7	260.3	19.53	6	1.63
20190322_Populatior Track	103522	154.92	205.08	0.761	3	0.254
20190322_Populatior Track	103528	94.05	265.95	11.79	6	0.983
20190322_Populatior Track	103534	139.06	220.94	7.99	6	0.666
20190322_Populatior Track	103542	44.84	44.84	5.29	8	0.294
20190322_Populatior Track	103550	11	11	10.49	8	0.583
20190322_Populatior Track	103553	34.84	34.84	0.241	3	0.0803
20190322_Populatior Track	103560	48.13	48.13	2.92	7	0.194
20190322_Populatior Track	103651	88.28	88.28	15.71	6	1.31
20190322_Populatior Track	103724	30.65	329.35	0.641	3	0.214
20190322_Populatior Track	103727	161.65	198.35	0.186	3	0.062
20190322_Populatior Track	103730	106.36	106.36	1.15	3	0.383

20190322_Populatior Track	103744	27.58	332.42	23.33	14	0.648
20190322_Populatior Track	103755	118.12	241.88	34.66	11	1.28
20190322_Populatior Track	103760	52.16	52.16	1.93	5	0.215
20190322_Populatior Track	103763	111.48	248.52	1.2	3	0.399
20190322_Populatior Track	103779	136.99	136.99	50.47	16	1.2
20190322_Populatior Track	103842	2.24	357.76	2.27	4	0.378
20190322_Populatior Track	103847	163.74	163.74	3.28	5	0.365
20190322_Populatior Track	103856	3.86	356.14	2.79	9	0.133
20190322_Populatior Track	103919	161.07	198.93	3.82	7	0.255
20190322_Populatior Track	103942	33.54	33.54	18.87	23	0.299
20190322_Populatior Track	103950	25.36	334.64	4.55	8	0.253
20190322_Populatior Track	103954	22.29	22.29	0.981	4	0.164
20190322_Populatior Track	103960	130.82	130.82	8.93	6	0.744
20190322_Populatior Track	104000	31.59	328.41	28.53	40	0.25
20190322_Populatior Track	104092	174.2	185.8	22.64	30	0.27
20190322_Populatior Track	104095	177.19	182.81	2.9	3	0.966

20190322_Populatior Track	116773	124.58	235.42	22.5	14	0.625
20190322_Populatior Track	116831	160.65	199.35	9.19	14	0.255
20190322_Populatior Track	116844	111.34	248.66	0.373	3	0.124
20190322_Populatior Track	116861	3.25	356.75	4.21	4	0.703
20190322_Populatior Track	117002	66.5	293.5	16.52	34	0.172
20190322_Populatior Track	117027	156.85	156.85	3.98	6	0.332
20190322_Populatior Track	117061	2.62	357.38	10.94	8	0.607
20190322_Populatior Track	117194	132.73	227.27	21.28	32	0.236
20190322_Populatior Track	117269	95.15	95.15	30.06	18	0.626
20190322_Populatior Track	117282	109.74	109.74	0.902	3	0.301
20190322_Populatior Track	117336	26.39	26.39	23.29	13	0.706
20190322_Populatior Track	117465	7.02	7.02	65.12	31	0.749
20190322_Populatior Track	117490	13.04	13.04	11.92	6	0.993
20190322_Populatior Track	117548	79.85	79.85	15.9	14	0.442
20190322_Populatior Track	117565	53.28	53.28	0.91	4	0.152
20190322_Populatior Track	117644	36.97	36.97	14.3	19	0.28

20190322_Populatior Track	117665	174.96	185.04	9.2	5	1.02
20190322_Populatior Track	117711	11.4	11.4	11.04	11	0.409
20190322_Populatior Track	118116	165.94	165.94	1.44	3	0.481
20190322_Populatior Track	118150	7.68	7.68	14.39	8	0.8
20190322_Populatior Track	118183	53.58	53.58	5.9	8	0.328
20190322_Populatior Track	118204	7.2	352.8	7.59	5	0.843
20190322_Populatior Track	118233	165.61	165.61	8.77	7	0.584
20190322_Populatior Track	118254	162.78	162.78	3.9	5	0.433
20190322_Populatior Track	118384	20.04	339.96	60.61	31	0.697
20190322_Populatior Track	118467	51.16	308.84	17.7	20	0.328
20190322_Populatior Track	118923	11.09	348.91	2.12	3	0.705
20190322_Populatior Track	118944	62.43	62.43	1.24	5	0.138
20190322_Populatior Track	118965	149.01	210.99	4.81	5	0.534
20190322_Populatior Track	119045	42.31	317.69	7.81	13	0.237
20190322_Populatior Track	119120	25.54	334.46	46.94	18	0.978
20190322_Populatior Track	119174	140.76	219.24	5.95	13	0.18
20190322_Populatior Track	119199	4.82	4.82	1.61	6	0.134
20190322_Populatior Track	119212	177.73	182.27	1.81	3	0.605
20190322_Populatior Track	119271	66.26	66.26	9.88	14	0.275
20190322_Populatior Track	119292	3.81	356.19	3.71	5	0.413
20190322_Populatior Track	119304	28.73	331.27	0.298	3	0.0994
20190322_Populatior Track	119321	6.25	6.25	1.94	4	0.323
20190322_Populatior Track	119333	4.05	355.95	3.63	3	1.21
20190322_Populatior Track	119359	168.51	168.51	12.36	6	1.03
20190322_Populatior Track	119371	64	296	0.28	3	0.0935
20190322_Populatior Track	119384	157.17	157.17	2.24	3	0.747
20190322_Populatior Track	119417	177.81	182.19	13.43	8	0.746
20190322_Populatior Track	119447	46.87	46.87	3.1	7	0.207
20190322_Populatior Track	119468	172.12	172.12	3.72	5	0.413
20190322_Populatior Track	119501	6.28	6.28	21.75	8	1.21
20190322_Populatior Track	119514	120.32	239.68	0.163	3	0.0544
20190322_Populatior Track	119526	10.16	349.84	3.22	3	1.07
20190322_Populatior Track	119543	46.23	46.23	2.03	4	0.338
20190322_Populatior Track	119581	180	180	6.94	9	0.33

20190322_Populatior Track	119660	43.71	316.29	31.99	19	0.627
20190322_Populatior Track	119673	64.97	64.97	0.784	3	0.261
20190322_Populatior Track	119736	8.01	8.01	6.05	15	0.155
20190322_Populatior Track	119769	5.23	5.23	17.15	8	0.953
20190322_Populatior Track	119786	14.42	345.58	1.31	4	0.218
20190322_Populatior Track	119799	177.26	177.26	0.127	3	0.0425
20190322_Populatior Track	119811	0.439	0.439	0.769	3	0.256
20190322_Populatior Track	119857	121.21	121.21	12.52	11	0.464
20190322_Populatior Track	119870	177.36	182.64	0.493	3	0.164
20190322_Populatior Track	119887	8.6	8.6	1.48	4	0.247
20190322_Populatior Track	119899	64.31	64.31	0.195	3	0.065
20190322_Populatior Track	120025	95.99	264.01	41.62	30	0.495
20190322_Populatior Track	120042	5.78	354.22	6.96	4	1.16
20190322_Populatior Track	178105	38.12	38.12	10.55	15	0.27
20190322_Populatior Track	178147	22.63	22.63	11.55	10	0.481
20190322_Populatior Track	178168	126.42	233.58	4.6	5	0.511
20190322_Populatior Track	178201	151.28	151.28	4.08	8	0.226
20190322_Populatior Track	178235	54.15	305.85	3.64	8	0.202
20190322_Populatior Track	178256	37.9	37.9	4.02	5	0.447
20190322_Populatior Track	178281	69.04	69.04	1.46	6	0.121
20190322_Populatior Track	178294	123.65	236.35	2.8	3	0.931
20190322_Populatior Track	178306	140.83	219.17	3.3	3	1.1
20190322_Populatior Track	178319	0.495	359.51	3.36	3	1.12
20190322_Populatior Track	178705	6.94	353.06	33.99	19	0.666
20190322_Populatior Track	178718	6.26	353.74	0.219	3	0.073
20190322_Populatior Track	178731	123.48	123.48	0.615	3	0.205
20190322_Populatior Track	178743	48.4	48.4	1.48	3	0.497
20190322_Populatior Track	178890	167.4	192.6	1.26	3	0.423
20190322_Populatior Track	178907	6.07	353.93	2.94	4	0.49
20190322_Populatior Track	178920	47.55	47.55	0.549	3	0.183
20190322_Populatior Track	179016	6.82	6.82	15.2	23	0.241
20190322_Populatior Track	179033	18.94	18.94	2.68	4	0.446

20190322_Populatior Track	179092	75.59	284.41	15.95	14	0.443
20190322_Populatior Track	179105	136.02	136.02	2.62	3	0.866
20190322_Populatior Track	179117	59	301	1.95	3	0.649
20190322_Populatior Track	179138	172.7	172.7	1.18	5	0.131
20190322_Populatior Track	179155	54.5	54.5	1.09	4	0.176
20190322_Populatior Track	179180	7.82	7.82	3.18	6	0.265
20190322_Populatior Track	179193	146.46	146.46	2.34	3	0.778
20190322_Populatior Track	179210	142.96	142.96	0.975	4	0.162
20190322_Populatior Track	179281	140.64	219.36	24.01	14	0.667
20190322_Populatior Track	179311	4.91	355.09	1.16	3	0.388
20190322_Populatior Track	179336	9.43	350.57	2.2	6	0.183
20190322_Populatior Track	179370	177.45	182.55	6.67	5	0.742
20190322_Populatior Track	179399	166.5	193.5	9.46	7	0.631
20190322_Populatior Track	179488	131.1	228.9	31.74	21	0.555
20190322_Populatior Track	179576	81.35	278.65	22.08	21	0.386
20190322_Populatior Track	179589	67.07	292.93	1.03	3	0.342
20190322_Populatior Track	179610	124.82	235.18	6.73	5	0.748
20190322_Populatior Track	179698	21.24	21.24	37.98	21	0.664
20190322_Populatior Track	179711	30.88	30.88	0.707	3	0.236
20190322_Populatior Track	179791	27.68	332.32	63.71	19	1.24
20190322_Populatior Track	179812	166.64	193.36	5.02	5	0.558
20190322_Populatior Track	179909	176.48	176.48	0.848	3	0.282
20190322_Populatior Track	179942	0.348	359.65	9.13	8	0.507
20190322_Populatior Track	179976	74.32	285.68	2.35	5	0.262
20190322_Populatior Track	179993	157.96	202.04	3.45	4	0.575
20190322_Populatior Track	180044	131.67	131.67	2.53	3	0.844
20190322_Populatior Track	180103	20.2	20.2	10.47	5	1.16
20190322_Populatior Track	180115	116.69	116.69	1.86	3	0.619

graunule s	nocodazol	0.494408
	sem	0.253053

granule speed EB3 colchicine

Item Name	Populatior Type	Track ID	Angle (deg	Bearing (d	Length (µn	Time Span	Track Velocity (µm/sec)
20190319_	Populatior Track		33872	170.78	170.78	7.69	13	0.233
20190319_	Populatior Track	33876	7.22	7.22	0.964	4	0.16
20190319_	Populatior Track	33879	169.27	169.27	1.24	3	0.414
20190319_	Populatior Track	33884	99.88	99.88	1.64	5	0.183
20190319_	Populatior Track	33889	159.2	200.8	1.47	5	0.164
20190319_	Populatior Track	33893	62.45	297.55	1.45	4	0.242
20190319_	Populatior Track	33897	76.82	76.82	1.03	4	0.171
20190319_	Populatior Track	33903	91.4	91.4	1.84	6	0.153
20190319_	Populatior Track	33908	125.55	125.55	2.32	5	0.258
20190319_	Populatior Track	33919	104.85	104.85	3.52	11	0.13
20190319_	Populatior Track	33922	149.62	149.62	0.833	3	0.278
20190319_	Populatior Track	33925	99.72	260.28	0.942	3	0.314
20190319_	Populatior Track	33929	10.1	349.9	0.648	4	0.108
20190319_	Populatior Track	33932	178.87	178.87	0.188	3	0.0627
20190319_	Populatior Track	33938	150.88	150.88	2.41	6	0.201
20190319_	Populatior Track	33944	169.3	169.3	4.13	6	0.344
20190319_	Populatior Track	33949	110.33	249.67	0.986	5	0.11
20190319_	Populatior Track	34108	171.39	188.61	1.5	3	0.5
20190319_	Populatior Track	34111	119.34	119.34	1.05	3	0.351
20190319_	Populatior Track	34124	87.13	272.87	3.27	13	0.0992
20190319_	Populatior Track	34127	31.77	31.77	0.286	3	0.0954
20190319_	Populatior Track	34130	153.27	206.73	0.202	3	0.0673
20190319_	Populatior Track	34133	42.04	317.96	0.75	3	0.25
20190319_	Populatior Track	34138	153.44	153.44	1.57	5	0.174
20190319_	Populatior Track	34141	7.63	7.63	0.353	3	0.118
20190319_	Populatior Track	34149	29.65	29.65	3.88	8	0.215
20190319_	Populatior Track	34158	125.84	234.16	3.44	9	0.164
20190319_	Populatior Track	34162	47.77	312.23	1.03	4	0.172
20190319_	Populatior Track	34175	23.15	336.85	4.33	13	0.131
20190319_	Populatior Track	34181	55.19	55.19	2.32	6	0.193
20190319_	Populatior Track	34187	23.33	336.67	0.869	6	0.0724
20190319_	Populatior Track	34199	9.34	9.34	13.7	12	0.457
20190319_	Populatior Track	34203	69.01	290.99	7.17	4	1.19

20190319_Populatior Track	34271	28.23	331.77	0.434	4	0.0724
20190319_Populatior Track	34282	5.05	354.95	6.19	11	0.229
20190319_Populatior Track	34289	126.18	233.82	32.06	12	0.971
20190319_Populatior Track	34294	3.93	3.93	1.12	3	0.374
20190319_Populatior Track	36291	96.84	96.84	0.519	3	0.173
20190319_Populatior Track	36298	26.2	333.8	0.168	3	0.0559
20190319_Populatior Track	36305	171.93	171.93	0.707	3	0.236
20190319_Populatior Track	36367	144.08	215.92	19.98	27	0.266
20190319_Populatior Track	36429	3.91	3.91	14.19	27	0.189
20190319_Populatior Track	36436	92.72	92.72	1.13	3	0.378
20190319_Populatior Track	36445	97.93	97.93	0.644	4	0.107
20190319_Populatior Track	36461	124.1	124.1	4.17	7	0.278
20190319_Populatior Track	36468	19.25	19.25	0.916	3	0.305
20190319_Populatior Track	36475	17.22	17.22	0.468	3	0.156
20190319_Populatior Track	36482	67.04	67.04	0.131	3	0.0435
20190319_Populatior Track	36493	77.95	77.95	1.25	5	0.139
20190319_Populatior Track	36525	56.69	303.31	3.56	14	0.0988
20190319_Populatior Track	36532	88.41	88.41	1.2	3	0.4
20190319_Populatior Track	36541	109.89	250.11	1	4	0.167
20190319_Populatior Track	36548	17.21	342.79	0.751	3	0.25
20190319_Populatior Track	36557	145.42	214.58	1.34	4	0.223
20190319_Populatior Track	36589	90.79	90.79	11.17	14	0.31
20190319_Populatior Track	36596	9.97	9.97	1.4	3	0.467
20190319_Populatior Track	36882	29.01	29.01	4.49	3	1.49
20190319_Populatior Track	36893	136.87	223.13	5.24	5	0.583
20190319_Populatior Track	36900	162.04	162.04	1.63	3	0.543
20190319_Populatior Track	36907	159.89	200.11	0.383	3	0.128
20190319_Populatior Track	36926	154	154	5.98	8	0.332
20190319_Populatior Track	36933	77.64	282.36	0.453	3	0.151
20190319_Populatior Track	36939	96.07	96.07	5.26	3	1.75
20190319_Populatior Track	36946	110.42	110.42	0.595	3	0.198
20190319_Populatior Track	36953	106.9	106.9	0.612	3	0.204

20190319_Populatior Track	36960	52.29	307.71	2.22	3	0.74
20190319_Populatior Track	36979	150.24	150.24	4.73	8	0.263
20190319_Populatior Track	36986	22.43	22.43	0.413	3	0.138
20190319_Populatior Track	37020	124.73	124.73	11.34	15	0.291
20190319_Populatior Track	37039	115.78	115.78	2.82	8	0.157
20190319_Populatior Track	37046	82.88	277.12	1.79	3	0.595
20190319_Populatior Track	37205	150.45	150.45	3.34	6	0.278
20190319_Populatior Track	37219	122.75	122.75	4.64	6	0.386
20190319_Populatior Track	37226	27.29	332.71	0.646	3	0.215
20190319_Populatior Track	37268	78.93	78.93	17.45	18	0.364
20190319_Populatior Track	37279	171.04	171.04	1.42	5	0.158
20190319_Populatior Track	37286	38.89	321.11	2.69	3	0.898
20190319_Populatior Track	37307	46.01	46.01	9.68	9	0.461
20190319_Populatior Track	37316	119.52	119.52	2.66	4	0.444
20190319_Populatior Track	37323	142.59	142.59	3.8	3	1.27
20190319_Populatior Track	37381	141.84	218.16	27.3	25	0.396
20190319_Populatior Track	37395	120.98	120.98	1.77	6	0.147
20190319_Populatior Track	37398	21.53	21.53	11.49	11	0.383

20190319_Populatior Track	42592	16.49	16.49	0.179	3	0.497
20190319_Populatior Track	42607	154.5	154.5	1.57	6	0.131
20190319_Populatior Track	42617	105.83	105.83	0.758	4	0.126
20190319_Populatior Track	42633	70.93	70.93	3.6	6	0.3
20190319_Populatior Track	42697	108.49	108.49	18.17	25	0.263
20190319_Populatior Track	42708	172.47	172.47	1.79	4	0.298
20190319_Populatior Track	42715	93.03	93.03	3.43	3	1.15
20190319_Populatior Track	42723	94.63	94.63	4.89	3	1.63
20190319_Populatior Track	42731	23.43	336.57	0.973	3	0.324
20190319_Populatior Track	42754	140.03	140.03	12.54	9	0.597
20190319_Populatior Track	42780	175.86	184.14	17.27	7	1.15
20190319_Populatior Track	42834	9.58	9.58	29.64	21	0.52
20190319_Populatior Track	42850	87.65	272.35	2.85	4	0.316
20190319_Populatior Track	42868	34.45	325.55	9.12	9	0.38

20190319_	Populatior Track	42878	70.25	289.75	15.3	13	0.464
20190319_	Populatior Track	42886	76.42	283.58	0.828	3	0.276
20190319_	Populatior Track	42930	105.48	254.52	27.65	17	0.614
20190319_	Populatior Track	42946	50.83	309.17	12.09	6	1.01
20190319_	Populatior Track	42969	130.12	130.12	16.35	9	0.779
20190319_	Populatior Track	42979	48.04	311.96	4.85	4	0.81
20190319_	Populatior Track	42987	143.35	143.35	3.95	3	1.32
20190319_	Populatior Track	43008	42.13	317.87	9.14	8	0.502
20190319_	Populatior Track	43015	167.83	192.17	1.44	3	0.481
20190327_	Populatior Track	43085	23.78	23.78	5.26	4	0.585
20190327_	Populatior Track	43095	4.96	4.96	6.81	9	0.284
20190327_	Populatior Track	43128	134.68	225.32	1.75	3	0.385
20190327_	Populatior Track	43133	34.29	34.29	0.543	4	0.181

granule sp colchicine	0.382685
sem	0.241878

granule speed EB3 taxol

Item Name	Populatior Type	Track ID	Angle (deg	Bearing (d	Length (µn	Time Span	Track Velocity (µm/sec)
20190322_	Populatior Track	86186	10.16	349.84	2.63	7	0.175
20190322_	Populatior Track	86189	151.45	208.55	0.705	3	0.235
20190322_	Populatior Track	86194	161.35	198.65	1.41	5	0.157
20190322_	Populatior Track	86208	23.31	23.31	9.45	14	0.263
20190322_	Populatior Track	86211	128.71	128.71	0.972	3	0.324
20190322_	Populatior Track	86214	93.82	266.18	0.547	3	0.183
20190322_	Populatior Track	86217	65.7	65.7	0.615	3	0.205
20190322_	Populatior Track	86221	63.42	296.58	0.485	4	0.0809
20190322_	Populatior Track	86233	178.99	181.01	6.63	12	0.221
20190322_	Populatior Track	86242	147.74	147.74	3.51	9	0.167
20190322_	Populatior Track	86248	4.63	4.63	1.36	6	0.114
20190322_	Populatior Track	86264	43.18	43.18	14.42	16	0.343
20190322_	Populatior Track	86285	58.68	301.32	6.06	21	0.106
20190322_	Populatior Track	86288	101.3	258.7	1.83	3	0.611

20190322_Populatior Track	86292	112.02	112.02	1.66	4	0.276
20190322_Populatior Track	86296	120.59	120.59	2.32	4	0.387
20190322_Populatior Track	86299	109.34	109.34	0.368	3	0.123
20190322_Populatior Track	86318	60.91	60.91	14.42	19	0.283
20190322_Populatior Track	86333	46.57	313.43	6.67	15	0.171
20190322_Populatior Track	86336	7.07	7.07	1.85	3	0.0618
20190322_Populatior Track	86343	165.92	165.92	5.26	7	0.35
20190322_Populatior Track	86347	46.5	46.5	0.666	4	0.111
20190322_Populatior Track	86350	167.89	192.11	0.368	3	0.123
20190322_Populatior Track	86353	69.26	290.74	0.961	3	0.32
20190322_Populatior Track	86357	146.21	213.79	2.32	4	0.387
20190322_Populatior Track	86360	84.7	84.7	0.512	3	0.17
20190322_Populatior Track	86363	124.83	235.17	1.37	3	0.456
20190322_Populatior Track	86366	26.59	26.59	0.284	3	0.0947
20190322_Populatior Track	86375	175.62	184.38	2.4	6	0.2
20190322_Populatior Track	86378	106.39	253.61	0.923	3	0.308
20190322_Populatior Track	86385	24.05	24.05	2.39	7	0.159
20190322_Populatior Track	86392	169.96	190.04	3.12	7	0.208
20190322_Populatior Track	86408	111.18	248.82	5.69	16	0.136
20190322_Populatior Track	86411	57.49	302.51	1.03	3	0.342
20190322_Populatior Track	86414	99.5	99.5	0.375	3	0.125
20190322_Populatior Track	86417	52.63	52.63	0.249	3	0.0831
20190322_Populatior Track	86420	34.64	34.64	0.434	3	0.145
20190322_Populatior Track	86424	27.55	27.55	0.935	4	0.156
20190322_Populatior Track	86432	20.97	20.97	3.5	8	0.194
20190322_Populatior Track	86441	3.15	356.85	3.74	9	0.178
20190322_Populatior Track	86451	143.25	216.75	5.64	10	0.235
20190322_Populatior Track	86454	161.24	161.24	1.12	3	0.375
20190322_Populatior Track	86463	150.42	150.42	3.28	6	0.274
20190322_Populatior Track	86467	161.59	198.41	0.912	4	0.152
20190322_Populatior Track	86470	149	149	0.56	3	0.187
20190322_Populatior Track	86473	167.09	167.09	0.599	3	0.199
20190322_Populatior Track	86476	36.4	36.4	0.0951	3	0.0317
20190322_Populatior Track	86479	128.04	231.96	1.56	3	0.521

20190322_Populatior Track	86482	167.53	167.53	0.168	3	0.0561
20190322_Populatior Track	86485	73.6	286.4	1.97	3	0.657
20190322_Populatior Track	86491	94.66	265.34	0.0947	3	0.0316
20190322_Populatior Track	86508	70.06	289.94	7.67	17	0.17
20190322_Populatior Track	86526	173.53	173.53	8.25	18	0.172
20190322_Populatior Track	86532	1.62	1.62	3.82	6	0.318
20190322_Populatior Track	86535	119.96	119.96	1.87	3	0.622
20190322_Populatior Track	86538	8.77	8.77	2.1	3	0.69
20190322_Populatior Track	86541	163.25	196.75	1.99	3	0.663
20190322_Populatior Track	86544	115.06	244.94	0.944	3	0.315
20190322_Populatior Track	86549	71.41	71.41	1.48	5	0.165
20190322_Populatior Track	86552	41.41	318.59	0.689	3	0.23
20190322_Populatior Track	86557	76.81	283.19	3.2	5	0.356
20190322_Populatior Track	86564	38.25	38.25	3.36	7	0.224
20190322_Populatior Track	86568	11.9	11.9	1.79	4	0.298
20190322_Populatior Track	86574	65.95	65.95	2.17	6	0.181
20190322_Populatior Track	86577	17.85	342.15	0.529	3	0.176
20190322_Populatior Track	86585	49.91	310.09	6.41	8	0.356
20190322_Populatior Track	86589	36.84	36.84	0.381	4	0.0635
20190322_Populatior Track	86592	30.85	30.85	0.43	3	0.143
20190322_Populatior Track	86598	27.83	27.83	1.45	6	0.12
20190322_Populatior Track	86605	22.66	337.34	2.82	7	0.188
20190322_Populatior Track	86610	19.35	340.65	2.18	5	0.242
20190322_Populatior Track	86616	116.25	243.75	1.68	6	0.14
				avg		
20190322_Populatior Track	96331	154.98	154.98	3.21	9	0.153
20190322_Populatior Track	96356	100.59	100.59	2.93	7	0.195
20190322_Populatior Track	96377	31.57	328.43	1.84	3	0.613
20190322_Populatior Track	96388	9.17	350.83	0.248	3	0.0827
20190322_Populatior Track	96406	113.94	113.94	2.5	5	0.278
20190322_Populatior Track	96417	79.3	79.3	0.449	3	0.15
20190322_Populatior Track	96432	57.46	57.46	1.4	4	0.234
20190322_Populatior Track	96446	116.11	243.89	1.15	4	0.192

20190322_Populatior Track	96464	28.66	28.66	1.78	5	0.198
20190322_Populatior Track	96475	159.78	200.22	0.626	3	0.209
20190322_Populatior Track	96486	146.76	213.24	1.1	3	0.367
20190322_Populatior Track	96536	35.44	35.44	6.35	14	0.176
20190322_Populatior Track	96551	170.76	189.24	0.555	4	0.0925
20190322_Populatior Track	96572	162.56	197.44	2.71	6	0.226
20190322_Populatior Track	96587	75.62	75.62	0.897	4	0.149
20190322_Populatior Track	96598	135.79	224.21	0.0376	3	0.0125
20190322_Populatior Track	96609	29.15	330.85	0.636	3	0.212
20190322_Populatior Track	96623	164.59	195.41	2.34	4	0.391
20190322_Populatior Track	96641	109	109	2.27	5	0.252
20190322_Populatior Track	96710	36.26	36.26	6.83	19	0.134
20190322_Populatior Track	96724	48.25	48.25	1	4	0.167
20190322_Populatior Track	96764	112.97	112.97	5.55	11	0.205
20190322_Populatior Track	96778	175.23	184.77	0.992	4	0.165
20190322_Populatior Track	96807	40.38	40.38	4.34	8	0.241
20190322_Populatior Track	96822	106.93	106.93	0.857	4	0.143
20190322_Populatior Track	96844	143.49	216.51	0.881	3	0.294
20190322_Populatior Track	96854	176.19	176.19	0.848	3	0.283
20190322_Populatior Track	97032	9.07	350.93	1.55	3	0.516
20190322_Populatior Track	97046	141.62	141.62	1.05	4	0.175
20190322_Populatior Track	97057	166.99	166.99	1.56	3	0.521
20190322_Populatior Track	97071	66.65	293.35	1.52	4	0.254
20190322_Populatior Track	97082	101	101	0.25	3	0.0834
20190322_Populatior Track	97093	101.16	258.84	0.419	3	0.14
20190322_Populatior Track	97108	27.65	27.65	2.82	4	0.47
20190322_Populatior Track	97136	9.74	9.74	2.74	5	0.304
20190322_Populatior Track	97155	62.2	62.2	3.05	5	0.339
20190322_Populatior Track	97169	160.48	199.52	0.632	4	0.105
20190322_Populatior Track	97234	25.48	334.52	12.58	18	0.262
20190322_Populatior Track	97245	22.11	337.89	1.3	3	0.434
20190322_Populatior Track	97256	116.24	116.24	0.263	3	0.0877
20190322_Populatior Track	97274	8.69	8.69	3.48	5	0.386
20190322_Populatior Track	97296	133.8	226.2	1.49	6	0.124

20190322_Populatior Track	97307	156.15	203.85	0.551	3	0.183
20190322_Populatior Track	97317	137.66	137.66	0.586	3	0.195
20190322_Populatior Track	97332	42.99	317.01	1.35	4	0.225
20190322_Populatior Track	97343	109.78	109.78	0.685	3	0.228
20190322_Populatior Track	97354	80.82	279.18	0.2	3	0.0665
20190322_Populatior Track	97368	90.46	90.46	2.68	4	0.447
20190322_Populatior Track	97379	92.21	267.79	1.63	3	0.544
20190322_Populatior Track	97390	86.89	273.11	1.98	3	0.661
20190322_Populatior Track	97401	53.41	306.59	0.421	3	0.14
20190322_Populatior Track	97412	16.88	16.88	0.389	3	0.13
20190322_Populatior Track	97422	105.63	105.63	0.523	3	0.174
20190322_Populatior Track	97433	8.55	351.45	0.816	3	0.272
20190322_Populatior Track	97466	127.15	232.85	6.49	9	0.309
20190322_Populatior Track	97477	83.94	276.06	0.29	3	0.0966
20190322_Populatior Track	97502	21.54	338.46	5.7	7	0.38
20190322_Populatior Track	97513	142.33	142.33	0.309	3	0.103
20190322_Populatior Track	97528	92.9	92.9	2.35	4	0.392
20190322_Populatior Track	97557	149.16	210.84	4.63	8	0.257
20190322_Populatior Track	97575	100.23	100.23	2.99	5	0.332
20190322_Populatior Track	97586	178.85	181.15	0.453	3	0.151
20190322_Populatior Track	97702	102.4	102.4	15.54	32	0.173
20190322_Populatior Track	97716	155.12	155.12	1.13	4	0.189
20190322_Populatior Track	97778	75.03	75.03	5.45	17	0.121
20190322_Populatior Track	97789	49.96	310.04	0.0758	3	0.0253
20190322_Populatior Track	97799	145.65	214.35	0.624	3	0.208
20190322_Populatior Track	97963	169.52	190.48	1.47	5	0.163
20190322_Populatior Track	97974	176.7	183.3	0.176	3	0.0587
20190322_Populatior Track	97984	44.32	315.68	0.233	3	0.0778
20190322_Populatior Track	98090	1.7	1.7	8.43	29	0.104
20190322_Populatior Track	98195	142.86	217.14	14.25	29	0.176
20190322_Populatior Track	98206	79.56	280.44	0.293	3	0.0977
20190322_Populatior Track	98217	80.06	80.06	0.858	3	0.286
20190322_Populatior Track	98253	131.86	228.14	5.87	10	0.244
20190322_Populatior Track	98264	67.86	67.86	0.9	3	0.3

20190322_Populatior Track	98351	31.32	31.32	3.16	7	0.211
20190322_Populatior Track	98373	172.91	172.91	6.23	6	0.519
20190322_Populatior Track	98384	140.8	219.2	0.289	3	0.0964
20190322_Populatior Track	98406	103.44	103.44	5.09	6	0.424
20190322_Populatior Track	98515	102	102	14.01	30	0.167
20190322_Populatior Track	98624	65.44	65.44	16.5	30	0.196
20190322_Populatior Track	98635	160.37	160.37	0.524	3	0.174
20190322_Populatior Track	98656	86.98	86.98	4.06	6	0.338
20190322_Populatior Track	98744	42.87	317.13	14.26	24	0.216
20190322_Populatior Track	98755	132.34	132.34	1.15	3	0.384
20190322_Populatior Track	98787	88.96	271.04	7.36	9	0.351
20190322_Populatior Track	98805	91.05	268.95	1.61	5	0.179
20190322_Populatior Track	98816	145.7	145.7	0.78	3	0.26
20190322_Populatior Track	98849	107.87	252.13	6.56	9	0.312
20190322_Populatior Track	98936	97.66	262.34	18.76	24	0.284
20190322_Populatior Track	98947	135.54	224.46	0.674	3	0.225
20190322_Populatior Track	98966	152.61	207.39	4.4	5	0.489
					avg	
20190322_Populatior Track	108827	74.8	285.2	7.53	13	0.228
20190322_Populatior Track	108838	144.26	215.74	0.23	3	0.0765
20190322_Populatior Track	108849	62.14	62.14	0.263	3	0.0877
20190322_Populatior Track	108922	132.5	227.5	8.17	20	0.151
20190322_Populatior Track	108937	62.96	62.96	0.73	4	0.122
20190322_Populatior Track	108992	54.49	305.51	0.419	4	0.0698
20190322_Populatior Track	109003	99.56	99.56	0.347	3	0.116
20190322_Populatior Track	109014	0.368	359.63	0.295	3	0.0983
20190322_Populatior Track	109025	146.25	213.75	0.98	3	0.327
20190322_Populatior Track	109206	160.05	160.05	0.477	3	0.159
20190322_Populatior Track	109217	154.48	205.52	0.962	3	0.321
20190322_Populatior Track	109232	12.2	347.8	0.558	4	0.093
20190322_Populatior Track	109246	160.81	199.19	2.77	4	0.462
20190322_Populatior Track	109268	141.77	218.23	3.31	6	0.276
20190322_Populatior Track	109280	40.25	319.75	0.889	3	0.296

20190322_Populatior Track	109291	64.78	295.22	0.351	3	0.117
20190322_Populatior Track	109302	5.32	354.68	1.76	3	0.587
20190322_Populatior Track	109327	178.23	181.77	7.13	7	0.476
20190322_Populatior Track	109434	87.64	272.36	23.84	29	0.294
20190322_Populatior Track	109515	143.74	143.74	9.46	22	0.158
20190322_Populatior Track	109534	160.72	160.72	2.31	5	0.256
20190322_Populatior Track	109600	92.27	267.73	18.6	18	0.388
20190322_Populatior Track	109652	91.05	91.05	4.31	14	0.12
20190322_Populatior Track	109718	36.08	323.92	3.17	9	0.151
20190322_Populatior Track	109733	8.21	351.79	1.97	4	0.328
20190322_Populatior Track	109752	58.03	58.03	3.05	5	0.339
20190322_Populatior Track	109763	100.94	100.94	0.576	3	0.192
20190322_Populatior Track	109777	81.78	278.22	1.18	4	0.196
20190322_Populatior Track	109807	161.25	198.75	4.88	8	0.271
20190322_Populatior Track	109818	170.91	189.09	0.863	3	0.288
20190322_Populatior Track	109836	39.04	320.96	3.42	5	0.38
20190322_Populatior Track	109851	24.89	24.89	1.25	4	0.209
20190322_Populatior Track	109884	95.15	95.15	3.29	9	0.157
20190322_Populatior Track	109910	79.78	280.22	6.06	7	0.0104
20190322_Populatior Track	109925	99.27	260.73	1.12	4	0.186
20190322_Populatior Track	109944	155.67	155.67	3.38	5	0.376
20190322_Populatior Track	109962	134.44	225.56	2.03	5	0.225
20190322_Populatior Track	110010	144.81	215.19	6.67	13	0.202
20190322_Populatior Track	110051	16.49	16.49	4.73	11	0.175
20190322_Populatior Track	110069	179.07	179.07	4.55	5	0.506
20190322_Populatior Track	110084	76.72	283.28	0.561	4	0.0935
20190322_Populatior Track	110095	8.29	8.29	0.0876	3	0.0292
20190322_Populatior Track	110106	141.72	141.72	0.76	3	0.253
20190322_Populatior Track	110117	37.73	37.73	1.41	3	0.471
20190322_Populatior Track	110128	72.39	72.39	0.602	3	0.202
20190322_Populatior Track	110139	109.48	109.48	0.977	3	0.327
20190322_Populatior Track	110162	14.01	345.99	4.29	6	0.358
20190322_Populatior Track	110173	44.55	44.55	0.435	3	0.145
20190322_Populatior Track	110202	165.2	194.8	5.41	8	0.3

20190322_Populatior Track	110213	38.42	38.42	0.172	3	0.0574
20190322_Populatior Track	110224	63.98	63.98	0.821	3	0.273
20190322_Populatior Track	110235	116.35	243.65	1.52	3	0.504
20190322_Populatior Track	110247	157.07	202.93	0.189	3	0.0631
20190322_Populatior Track	110258	158.33	201.67	1.53	3	0.559
20190322_Populatior Track	110272	1.86	1.86	1.43	4	0.238
20190322_Populatior Track	110287	94.63	265.37	2.31	4	0.385
20190322_Populatior Track	110302	146.17	213.83	1.47	4	0.244
20190322_Populatior Track	110313	65.19	65.19	0.976	3	0.325
20190322_Populatior Track	110343	1.11	1.11	7.07	8	0.393
20190322_Populatior Track	110354	17.35	342.65	1.15	3	0.384
20190322_Populatior Track	110369	118.17	241.83	1.81	4	0.302
20190322_Populatior Track	110402	63.87	63.87	7.06	9	0.336
20190322_Populatior Track	110431	145.92	214.08	5.23	8	0.29
20190322_Populatior Track	110442	96.18	263.82	1.7	3	0.567
20190322_Populatior Track	110454	59.58	59.58	0.725	3	0.226
20190322_Populatior Track	110502	110.48	249.52	0.285	5	0.0151
20190322_Populatior Track	110524	150	210	0.821	3	0.274
				avg		
				granule sp taxol		0.244484
				sem		0.099559

Microtubule end speed

particle tracking for EB3-GFP transfected cells

avg unstim	0.516406	stim DMSO	0.571591
sem	0.016544	sem	0.033419

DMSO unstimulated

Item Name	Population	Type	Track ID	Angle (deg	Bearing (d	Length (µm	Time Span	Track Velocity (µm/sec)
20190315_	Population	Track	8870	38.83	38.83	9.431	8	0.629
20190315_	Population	Track	8875	159.95	200.05	2.371	6	0.158
20190315_	Population	Track	8889	64.18	64.18	4.201	4	0.28
20190315_	Population	Track	8897	30.55	329.45	3.504	4	0.234
20190315_	Population	Track	8911	9.6	9.6	12.971	6	0.865
20190315_	Population	Track	8914	167.46	192.54	11.726	5	0.977
20190315_	Population	Track	8920	87.02	272.98	9.252	5	0.771
20190315_	Population	Track	8924	122.81	237.19	5.891	3	0.491
20190315_	Population	Track	8929	8.37	351.63	0.519	5	0.0427
20190315_	Population	Track	8931	92.76	267.24	0.386	3	0.0643
20190315_	Population	Track	8934	35.65	324.35	5.033	3	0.84
20190315_	Population	Track	8947	49.52	310.48	1.701	5	0.564
20190315_	Population	Track	8948	7.74	7.74	0.175	6	0.0585
20190315_	Population	Track	8956	32.82	327.18	1.396	3	0.466
20190315_	Population	Track	8963	150.46	150.46	6.365	11	2.119
20190315_	Population	Track	8974	17.27	17.27	1.082	3	0.362
20190315_	Population	Track	8977	66.47	293.53	0.897	5	0.299
								0.542382
20190322_	Population	Track	18219	91.25	268.75	1.912	7	0.106
20190322_	Population	Track	18222	66.48	293.52	0.411	4	0.137
20190322_	Population	Track	18238	62.36	297.64	1.734	15	0.5413
20190322_	Population	Track	18242	64.14	295.86	0.475	3	0.0791
20190322_	Population	Track	18250	34.73	325.27	0.743	7	0.7413
20190322_	Population	Track	18253	67.94	292.06	0.474	3	0.158
20190322_	Population	Track	18261	140.67	140.67	0.96	7	0.7533

20190322_ Populatior Track	18269	155.3	204.7	0.962	7	0.1535
20190322_ Populatior Track	18273	105.99	254.01	1.78	4	0.596
20190322_ Populatior Track	18277	99.03	99.03	1.689	4	1.418
20190322_ Populatior Track	18280	103.7	256.3	0.222	3	0.596
20190322_ Populatior Track	18283	100.88	259.12	0.392	4	0.758
20190322_ Populatior Track	18290	0.996	0.996	2.541	3	0.423
20190322_ Populatior Track	18293	160.16	160.16	0.106	3	0.0354
20190322_ Populatior Track	18297	44.15	44.15	4.696	6	0.785
20190322_ Populatior Track	18305	107.05	252.95	2.402	5	0.5669
20190322_ Populatior Track	18308	136.25	223.75	0.676	3	0.225
20190322_ Populatior Track	18312	138.03	221.97	5.228	4	0.873
						0.496989
20190322_ Populatior Track	22010	84.06	275.94	8.979	6	0.748
20190322_ Populatior Track	22011	3.75	3.75	0.825	3	0.275
20190322_ Populatior Track	22017	83.04	276.96	0.306	3	0.306
20190322_ Populatior Track	22018	72.03	72.03	0.739	3	0.246
20190322_ Populatior Track	22020	137.54	222.46	0.801	4	0.133
20190322_ Populatior Track	22021	93.28	93.28	30.542	9	1.454
20190322_ Populatior Track	22022	114.17	245.83	2.048	5	0.228
20190322_ Populatior Track	22027	41.18	41.18	11.674	9	0.556
20190322_ Populatior Track	22029	66.19	66.19	2.24	3	0.58
20190322_ Populatior Track	22033	105.99	254.01	2.787	4	0.464
20190322_ Populatior Track	22036	99.03	99.03	5.678	4	0.948
20190322_ Populatior Track	22042	103.7	256.3	0.319	3	0.556
20190322_ Populatior Track	22046	100.88	259.12	0.4	3	0.134
						0.509846
					0	0.542382
					0	0.496989
					0	0.509846
				avg unstim		0.516406
				sem		0.016544

DMSO stimulated

Item Name	Population Type	Track ID	Angle (deg	Bearing (d	Length (µn	Time Span	Track Velocity (µm/sec)
20190327_	Populatior Track	19965	165.75	194.25	6.916	4	1.152
20190327_	Populatior Track	19969	26.93	26.93	3.327	4	0.554
20190327_	Populatior Track	19972	15.06	344.94	0.308	3	0.103
20190327_	Populatior Track	19975	114.26	114.26	0.0354	3	0.0118
20190327_	Populatior Track	19982	94.81	265.19	9.133	7	0.609
20190327_	Populatior Track	19985	162.59	162.59	3.326	3	1.108
20190327_	Populatior Track	19989	95.18	264.82	4.854	4	0.809
20190327_	Populatior Track	19993	132.69	227.31	14.091	4	2.348
20190327_	Populatior Track	19996	48.22	48.22	2.422	5	0.807
20190327_	Populatior Track	19999	69.56	69.56	0.188	3	0.0626
20190327_	Populatior Track	20003	10.82	349.18	4.655	6	0.776
20190327_	Populatior Track	20006	158.8	201.2	0.156	3	0.0519
20190327_	Populatior Track	20024	166.4	166.4	7.726	17	0.161
20190327_	Populatior Track	20027	121.34	121.34	0.132	3	0.044
20190327_	Populatior Track	20030	78.59	281.41	0.969	5	0.323
20190327_	Populatior Track	20035	142.11	142.11	23.333	8	2.593
20190327_	Populatior Track	20038	105.4	254.6	0.361	3	0.12
20190327_	Populatior Track	20044	99.71	99.71	10.818	7	0.902
20190327_	Populatior Track	20047	16.03	343.97	0.194	3	0.0647
20190327_	Populatior Track	20050	157.55	202.45	2.003	9	0.668
20190327_	Populatior Track	20053	70.87	70.87	0.811	5	0.27
20190327_	Populatior Track	20056	116.12	243.88	0.268	3	0.0893
20190327_	Populatior Track	20059	99.07	260.93	0.411	4	0.137
							0.598448
20190327_	Populatior Track	23387	155.65	204.35	4.147	7	0.346
20190327_	Populatior Track	23390	167.41	192.59	2.217	4	0.738
20190327_	Populatior Track	23393	154.39	205.61	0.623	3	0.208
20190327_	Populatior Track	23396	34.71	34.71	2.262	3	0.754
20190327_	Populatior Track	23399	125.8	234.2	1.22	6	0.407
20190327_	Populatior Track	23403	101.39	258.61	0.84	4	0.14
20190327_	Populatior Track	23406	171.21	171.21	5.787	11	1.929

20190327_ Populatior Track	23410	3.73	3.73	2.647	4	0.441
20190327_ Populatior Track	23415	121	239	0.523	5	0.0581
20190327_ Populatior Track	23418	174.75	185.25	0.0797	3	0.0266
20190327_ Populatior Track	23421	56.29	303.71	2.678	3	0.893
20190327_ Populatior Track	23424	17.74	17.74	2.764	5	0.922
20190327_ Populatior Track	23427	172.56	187.44	0.188	3	0.0625
20190327_ Populatior Track	23430	125.35	234.65	0.46	3	0.153
20190327_ Populatior Track	23434	79.3	280.7	3.079	6	0.513
20190327_ Populatior Track	23437	58.37	58.37	2.613	9	0.871
20190327_ Populatior Track	23442	42.7	42.7	5.553	4	0.618
20190327_ Populatior Track	23445	148.5	211.5	0.946	3	0.315
20190327_ Populatior Track	23448	99.52	99.52	0.285	3	0.095
20190327_ Populatior Track	23451	75.42	284.58	0.124	4	0.0415
20190327_ Populatior Track	23455	116.14	243.86	0.986	5	0.164
20190327_ Populatior Track	23459	78.34	78.34	2.827	7	0.47
20190327_ Populatior Track	23463	94.36	265.64	3.721	4	0.62
20190327_ Populatior Track	23467	134.84	134.84	6.149	4	1.025
20190327_ Populatior Track	23471	74.69	285.31	5.258	4	0.876
20190327_ Populatior Track	23475	141.08	141.08	3.885	6	0.647
20190327_ Populatior Track	23478	114.57	245.43	0.373	3	0.124
20190327_ Populatior Track	23482	26.38	26.38	11.922	4	1.987
20190327_ Populatior Track	23486	117.98	242.02	0.276	4	0.0461
						0.534166
20190327_ Populatior Track	30639	95.32	264.68	12.737	6	2.123
20190327_ Populatior Track	30642	108.32	251.68	0.376	4	0.125
20190327_ Populatior Track	30645	64.29	64.29	5.377	7	1.792
20190327_ Populatior Track	30648	62.24	297.76	1.643	3	0.548
20190327_ Populatior Track	30652	51.37	308.63	2.477	3	0.825
20190327_ Populatior Track	30655	49.83	310.17	3.89	4	0.648
20190327_ Populatior Track	30659	72.98	72.98	2.293	3	0.765
20190327_ Populatior Track	30664	55.87	304.13	3.63	5	1.017
20190327_ Populatior Track	30667	158.16	201.84	0.228	4	0.0757
20190327_ Populatior Track	30670	150.88	209.12	0.247	3	0.0825

20190327_ Populatior Track	30673	148.04	211.96	0.341	3	0.114
20190327_ Populatior Track	30676	77.92	282.08	0.116	3	0.0387
20190327_ Populatior Track	30679	141.06	218.94	1.107	4	0.371
20190327_ Populatior Track	30683	129.02	230.98	0.488	5	0.162
20190327_ Populatior Track	30686	114.44	245.56	0.137	3	0.0455
						0.58216
						0.598448
						0.534166
						0.58216
					stim DMSC	0.571591
					sem	0.033419

Fig 3A Data: Kinesore effect on exocytosis

RBL-2H3 + kinesore

uM drug	Exocytosis	s.e.m.	t-test
0	100	0	na
6.25	76.01304	2.045871	0.307849
12.5	66.80606	4.688958	0.141246
25	52.08369	3.007153	0.056296
50	43.4927	6.392481	0.031597
100	23.61806	10.23525	0.010427

BMMC + kinesore

uM drug	Exocytosis	s.e.m.	t-test
0	100	0	na
6.25	92.36488	4.532258	0.449882
12.5	90.06766	5.103279	0.36881
25	87.4935	3.304649	0.27793
50	72.46899	5.52375	0.03586
100	56.08044	3.762601	0.008762

Trial1

Kinesore RBL-2H3	0	6.25	12.5	25	50	100	
Rest	1913	1667	1656	1789	1868	1238	360,460
Stim1	20883	19773	15086	10509	9616	3568	360,460
Stim2	18464	17986	16578	9837	8415	4807	360,460
Stim3	18548	18455	15492	9331	9707	4047	360,460
Lyse R	39447	37517	35972	26772	44417	34766	360,460
Lyse S1	23434	31850	27168	24750	36057	37028	360,460
Lyse S2	21728	33956	31701	24484	38807	43120	360,460
Lyse S3	21520	39729	43029	28048	45937	37624	360,460

Trial2

Kinesore RBL-2H3	0	6.25	12.5	25	50	100	
Rest	952	976	1094	948	1021	883	360,460
Stim1	23545	16688	10638	8527	7333	3583	360,460
Stim2	19710	15695	11136	8175	7521	3840	360,460
Stim3	21316	15717	12142	7659	7063	3285	360,460
Lyse R	46636	33515	32645	35177	29182	39609	360,460
Lyse S1	22306	23200	23528	23308	22802	34087	360,460
Lyse S2	20156	24447	23974	22595	22301	28837	360,460
Lyse S3	20425	25511	22592	23297	25068	34660	360,460

Trial3

Kinesore RBL-2H3	0	6.25	12.5	25	50	100	
Rest	1526	2149	1558	1484	1434	1816	360,460
Stim1	19673	11047	9696	6136	7136	6185	360,460
Stim2	11199	10140	10496	7286	6286	6541	360,460
Stim3	12548	11346	10732	8181	7181	5762	360,460
Lyse R	47935	38550	41478	42815	40104	37207	360,460
Lyse S1	33214	30080	32764	32760	28562	32967	360,460
Lyse S2	33457	31915	35158	32000	34151	33270	360,460
Lyse S3	36386	30651	36016	32227	35993	37103	360,460

Exo	0	6.25	12.5	25	50	100
Rest	4.63	4.25	4.40	6.26	4.04	3.44

Trial1

Kinesore BMMCs	0	6.25	12.5	25	50	100	
Rest	5074	4900	4970	4900	4581	3954	360,460
Stim1	26584	23040	22736	23040	21849	17015	360,460
Stim2	27792	21537	21997	21537	19057	15058	360,460
Stim3	25583	20841	24950	24841	19629	15061	360,460
Lyse R	29161	32293	31601	32293	29839	39163	360,460
Lyse S1	34077	27437	30964	33437	37582	40823	360,460
Lyse S2	33430	28450	27661	31450	29831	46616	360,460
Lyse S3	26800	27947	29138	30947	30811	32505	360,460

Trial2

Kinesore BMMCs	0	6.25	12.5	25	50	100	
Rest	5566	5341	5108	5341	5391	5201	360,460
Stim1	23292	24061	22718	22061	19521	15729	360,460
Stim2	25102	24845	25853	23845	18437	13651	360,460
Stim3	29547	27576	26784	23576	20908	15450	360,460
Lyse R	42743	29053	27215	29053	36253	44834	360,460
Lyse S1	28731	28988	28551	27988	32689	39111	360,460
Lyse S2	34167	29779	31174	29779	33617	34481	360,460
Lyse S3	35141	30520	34994	30520	34100	39966	360,460

Trial3

Kinesore BMMCs	0	6.25	12.5	25	50	100	
Rest	3507	3936	3883	4136	3799	4006	360,460
Stim1	34268	27879	27994	27879	21761	17111	360,460
Stim2	31721	25305	24870	24305	20496	17344	360,460
Stim3	32075	26370	25365	23370	19333	19225	360,460
Lyse R	30720	31054	31244	31054	37557	43952	360,460
Lyse S1	31383	28832	32098	28832	33265	41330	360,460
Lyse S2	30773	31945	29725	28945	37471	49357	360,460
Lyse S3	30390	30892	30033	30892	40915	38135	360,460

Exo	0	6.25	12.5	25	50	100
Rest	14.82	13.17	13.59	13.17	13.31	9.17

**Stim1**	47.12	38.30	35.70	29.81	21.05	8.79
**Stim2**	45.94	34.63	34.34	28.66	17.82	10.03
**Stim3**	46.29	31.72	26.47	24.96	17.44	9.71

Exo	**0**	**6.25**	**12.5**	**25**	**50**	**100**
**Rest**	2.00	2.83	3.24	2.62	3.38	2.18
**Stim1**	51.35	41.84	31.14	26.78	24.33	9.51
**Stim2**	49.44	39.10	31.72	26.57	25.22	11.75
**Stim3**	51.07	38.12	34.96	24.74	21.98	8.66

Exo	**0**	**6.25**	**12.5**	**25**	**50**	**100**
**Rest**	3.09	5.28	3.62	3.35	3.45	4.65
**Stim1**	37.20	26.86	22.84	15.78	19.99	15.80
**Stim2**	25.08	24.11	22.99	18.55	15.55	16.43
**Stim3**	25.64	27.02	22.96	20.25	16.63	13.44

avg - rest	**Exo kin1**	41.83	30.63	27.77	21.55	14.74	6.07
	**Exo kin2**	48.62	36.86	29.36	23.41	20.46	7.79
	**Exo kin3**	26.22	20.72	19.31	14.84	13.94	10.57
normalized	**Exo kin1**	100	73.2288	66.39519	51.51446	35.23463	14.51673
	**Exo kin2**	100	75.80605	60.38987	48.14423	42.09175	16.02808
	**Exo kin3**	100	79.00428	73.63313	56.5924	53.15173	40.30936
	**Exo kin avg**	100	76.01304	66.80606	52.08369	43.4927	23.61806
	**Exo kin sem**	0	2.045871	4.688958	3.007153	6.392481	10.23525

**Stim1**	43.82	45.64	42.34	40.80	36.76	29.42
**Stim2**	45.40	43.09	44.30	40.65	38.98	24.42
**Stim3**	48.84	42.72	46.13	44.53	38.92	31.66

Exo	**0**	**6.25**	**12.5**	**25**	**50**	**100**
**Rest**	11.52	15.53	15.80	15.53	12.95	10.39
**Stim1**	44.77	45.36	44.31	44.08	37.39	28.68
**Stim2**	42.35	45.48	45.33	44.47	35.42	28.36
**Stim3**	45.68	47.47	43.36	43.58	38.01	27.88

Exo	**0**	**6.25**	**12.5**	**25**	**50**	**100**
**Rest**	10.25	11.25	11.05	11.75	9.19	8.35
**Stim1**	52.20	49.16	46.59	49.16	39.55	29.28
**Stim2**	50.76	44.20	45.55	45.64	35.36	26.00
**Stim3**	51.35	46.05	45.79	43.07	32.09	33.52

avg - rest	**Exo kin1**	31.20	30.64	30.66	28.82	24.91	19.33
	**Exo kin2**	32.75	30.57	28.53	28.51	23.99	17.91
	**Exo kin3**	41.19	35.22	34.92	34.20	26.48	21.25
normalize	**Exo kin1**	100	98.2149	98.29052	92.36141	79.84745	61.95459
	**Exo kin2**	100	93.36615	87.12897	87.07673	73.27335	54.70386
	**Exo kin3**	100	85.51359	84.78348	83.04236	64.28617	51.58288
	**Exo kin avg**	100	92.36488	90.06766	87.4935	72.46899	56.08044
	**Exo kin sem**	0	4.532258	5.103279	3.304649	5.52375	3.762601

Data for Fig 3A

RBL-2H3 + kinesore

uM drug	tosis (norm	s.e.m.	t-test
0	100.00	0.00	na
6.25	76.01	2.05	0.307849
12.5	66.81	4.69	0.141246
25	52.08	3.01	0.056296
50	43.49	6.39	0.031597
100	23.62	10.24	0.010427

BMMC + kinesore

uM drug	tosis (norm	s.e.m.	t-test
0	100.00	0.00	na
6.25	92.36	4.53	0.449882
12.5	90.07	5.10	0.36881
25	87.49	3.30	0.27793
50	72.47	5.52	0.03586
100	56.08	3.76	0.008762

Fig 5B Raw Data: Effect of kinesin-1 KD on exocytosis

		avg	sd	sem	ttest vs WT	ttest vs sc
unstimula	wt	14.4147	4.57132	2.63933	na	0.502017
	sc	12.7619	3.78868	2.18746	0.50201701	na
	#479	13.4309	3.87742	2.2387	0.55712468	0.9253433
stimulatec	wt	100	0	0	na	0.62282
	sc	96.6784	14.9083	8.60754	0.88610957	na
	#479	62.2423	11.0071	6.35516	0.0042934	0.0287186

	Resting 1	Resting 2	Resting 3	Resting 4	Stim. 1	Stim. 2	Stim. 3	Stim. 4
WT	12.5159	20.309	12.0482337	12.78577	100	100	100	100
Scrambled	13.4835	15.5031	13.8027507	8.2581024	90.6537	97.6518	114.085	84.3228
#479	15.2229	17.1793	11.5935078	9.727792	60.2549	69.8651	68.6344	50.2147

Assay 1	Resting Supernatant 1	Resting Supernatant 2	Resting Supernatant 3	Stimulated Supernatant 1	Stimulated Supernatant 2	Stimulated Supernatant 3	Resting Lysate 1	Resting Lysate 2	Resting Lysate 3	Stimulated Lysate 1	Stimulated Lysate 2	Stimulated Lysate 3
WT	2162	2211	1765	14982	15221	16196	39950	41763	39508	25433	25281	23589
Scrambled	2247	2120	2013	12732	18485	11418	38308	39231	39177	27269	26342	24815
479	2642	3164	2464	12610	8424	9998	44999	43676	44375	36828	33311	31977

% exo=sup/(lys+sup)

	Assay 1	Resting Exocytosis 1	Resting Exocytosis 2	Resting Exocytosis 3	Stimulated Exocytosis 1	Stimulated Exocytosis 2	Stimulated Exocytosis 3	Resting Exocytosis Avg	Stimulated Exocytosis Avg	Resting Normalized to WT Stim	Stimulated Normalized to WT Stim
360,460	WT	5.13393	5.02797	4.2764	37.0704	37.5809	40.7088	4.81277	38.4534	12.5159	100
360,460	Scrambled	5.54062	5.12684	4.88711	31.8292	41.2363	31.5127	5.18486	34.8594	13.4835	90.6537
360,460	479	5.54564	6.75491	5.26057	25.5067	20.1845	23.8189	5.85371	23.17	15.2229	60.2549

Assay 3	Resting Supernatant 1	Resting Supernatant 2	Resting Supernatant 3	Stimulated Supernatant 1	Stimulated Supernatant 2	Stimulated Supernatant 3	Resting Lysate 1	Resting Lysate 2	Resting Lysate 3	Stimulated Lysate 1	Stimulated Lysate 2	Stimulated Lysate 3
WT	2940	3387	3278	14165	17079	16840	38348	39081	39124	26013	26098	27963
Scrambled	3032	2556	2458	15841	18499	20030	44113	44726	41528	32516	32780	28913
479	3451	2684	3297	12308	12089	15272	45956	43949	46895	38894	36796	36046

	Assay 2	Resting Exocytosis 1	Resting Exocytosis 2	Resting Exocytosis 3	Stimulated Exocytosis 1	Stimulated Exocytosis 2	Stimulated Exocytosis 3	Resting Exocytosis Avg	Stimulated Exocytosis Avg	Resting Normalized to WT Stim	Stimulated Normalized to WT Stim
360,460	WT	7.12071	7.97542	7.73077	35.2556	39.5558	37.5868	7.60897	37.4661	20.309	100
360,460	Scrambled	6.43122	5.40586	5.58814	32.7584	36.0752	40.9252	5.80841	36.5863	15.5031	97.6518
360,460	479	6.98484	5.75558	6.56878	24.0381	24.7295	29.7595	6.4364	26.1757	17.1793	69.8651

Assay 4	Resting Supernatant 1	Resting Supernatant 2	Resting Supernatant 3	Stimulated Supernatant 1	Stimulated Supernatant 2	Stimulated Supernatant 3	Resting Lysate 1	Resting Lysate 2	Resting Lysate 3	Stimulated Lysate 1	Stimulated Lysate 2	Stimulated Lysate 3
WT	1683	974	1833	12784	9796	9495	42863	48492	45453	29183	30921	27954
Scrambled	960	1916	1699	11484	10959	11666	38934	39222	41193	26890	26997	24422
479	1525	1059	1575	7063	8662	7606	42770	44437	43556	34695	37796	31879

	Assay 3	Resting Exocytosis 1	Resting Exocytosis 2	Resting Exocytosis 3	Stimulated Exocytosis 1	Stimulated Exocytosis 2	Stimulated Exocytosis 3	Resting Exocytosis Avg	Stimulated Exocytosis Avg	Resting Normalized to WT Stim	Stimulated Normalized to WT Stim
360,460	WT	3.77812	1.96903	3.87641	30.462	24.0587	25.3545	3.20785	26.6251	12.0482	100
360,460	Scrambled	2.40638	4.65749	3.96111	29.9265	28.8729	32.3265	3.67499	30.3753	13.8028	114.085
360,460	479	3.44283	2.32768	3.48984	16.9141	18.6448	19.263	3.08678	18.274	11.5935	68.6344

Assay 4	Resting Supernatant 1	Resting Supernatant 2	Resting Supernatant 3	Stimulated Supernatant 1	Stimulated Supernatant 2	Stimulated Supernatant 3	Resting Lysate 1	Resting Lysate 2	Resting Lysate 3	Stimulated Lysate 1	Stimulated Lysate 2	Stimulated Lysate 3
WT	1798	2061	1492	12597	11559	13005	38640	39920	39014	23498	24487	24137
Scrambled	1302	1603	1055	13356	14049	11891	45186	45782	46084	32966	32061	32658
479	1171	1852	1431	7667	6168	7902	41435	42886	45759	35334	35540	34602

	Assay 4	Resting Exocytosis 1	Resting Exocytosis 2	Resting Exocytosis 3	Stimulated Exocytosis 1	Stimulated Exocytosis 2	Stimulated Exocytosis 3	Resting Exocytosis Avg	Stimulated Exocytosis Avg	Resting Normalized to WT Stim	Stimulated Normalized to WT Stim
360,460	WT	4.44631	4.90936	3.6834	34.8996	32.0674	35.0143	4.34636	33.9937	12.7858	100
360,460	Scrambled	2.80072	3.38293	2.23806	28.833	30.4684	26.692	2.80724	28.6645	8.2581	84.3228
360,460	479	2.74844	4.13966	3.03242	17.8298	14.7885	18.5912	3.30684	17.0698	9.72779	50.2147

Fig 7A Raw Data: Relative expression of cargo adaptors

	relative expression to GAPDH	sem
JIP1	0.0164	0.0070
JIP2	0.1004	0.0722
JIP3	1.7682	0.8821
JIP4	1.1401	0.1532
PKHM	0.4314	0.0254
SKIP	0.8212	0.1671
Slp1	0.3868	0.3361
Slp2	1.4967	0.2739
Slp3	3.9342	0.4545
Slp4	0.0031	0.0012
Slp5	0.0288	0.0202

	Adaptors	CT value (	ΔCT (vs. G	-ΔCT	2^-ΔCT	ıalized (%)
Trial 1	GAPDH	12.89			1	100
	JIP1	24.72	11.83	-11.83	0.000275	0.027467
	JIP2	21.73	8.84	-8.84	0.002182	0.21822
	JIP3	17.87	4.98	-4.98	0.031686	3.168623
	JIP4	19.14	6.25	-6.25	0.013139	1.313901
	Plehkm1	20.75	7.86	-7.86	0.004304	0.430432
	SKIP	19.42	6.53	-6.53	0.010821	1.082117
	Slp1	19.63	6.74	-6.74	0.009355	0.93553
	Slp2	18.59	5.7	-5.7	0.019237	1.923663
	Slp3	17.31	4.42	-4.42	0.046714	4.671404
	Slp4	27.21	14.32	-14.32	4.89E-05	0.004889
	Slp5	23.55	10.66	-10.66	0.000618	0.061805
Trial 2	GAPDH	12.29			1	100
	JIP1	25.17	12.88	-12.88	0.000133	0.013266
	JIP2	23.43	11.14	-11.14	0.000443	0.044312

	JIP3	18.49	6.2	-6.2	0.013602	1.360235
	JIP4	18.66	6.37	-6.37	0.01209	1.209035
	Plehkm1	20.03	7.74	-7.74	0.004678	0.467765
	SKIP	19.62	7.33	-7.33	0.006215	0.621513
	Slp1	21.99	9.7	-9.7	0.001202	0.120229
	Slp2	18.45	6.16	-6.16	0.013985	1.398477
	Slp3	17.07	4.78	-4.78	0.036398	3.639792
	Slp4	27.4	15.11	-15.11	2.83E-05	0.002828
	Slp5	25.17	12.88	-12.88	0.000133	0.013266
Trial 3	GAPDH	11.98			1	100
	JIP1	25.49	13.51	-13.51	8.57E-05	0.008572
	JIP2	23.32	11.34	-11.34	0.000386	0.038576
	JIP3	18.99	7.01	-7.01	0.007759	0.775854
	JIP4	18.78	6.8	-6.8	0.008974	0.897421
	Plehkm1	19.96	7.98	-7.98	0.003961	0.396078
	SKIP	19.02	7.04	-7.04	0.007599	0.759887
	Slp1	21.88	9.9	-9.9	0.001047	0.104665
	Slp2	18.4	6.42	-6.42	0.011679	1.167851
	Slp3	16.82	4.84	-4.84	0.034915	3.491522
	Slp4	27.87	15.89	-15.89	1.65E-05	0.001647
	Slp5	25.1	13.12	-13.12	0.000112	0.011233
Average	GAPDH	12.38667			1	100
	JIP1	25.12667	12.74	-12.74	0.000164	0.016435
	JIP2	22.82667	10.44	-10.44	0.001004	0.10037
	JIP3	18.45	6.063333	-6.06333	0.017682	1.768237
	JIP4	18.86	6.473333	-6.47333	0.011401	1.140119
	Plehkm1	20.24667	7.86	-7.86	0.004314	0.431425
	SKIP	19.35333	6.966667	-6.96667	0.008212	0.821172
	Slp1	21.16667	8.78	-8.78	0.003868	0.386808
	Slp2	18.48	6.093333	-6.09333	0.014967	1.496664
	Slp3	17.06667	4.68	-4.68	0.039342	3.93424
	Slp4	27.49333	15.10667	-15.1067	3.12E-05	0.003121

Slp5	24.60667	12.22	-12.22	0.000288	0.028768

Fig 7D Raw Data: Relative levels of protein association with granule enriched fractions

	Kif5b avg	Kif5b sem	RMCP-II av	RMCP-II se	Slp3 avg	Slp3 sem
unstimulated	1	0	1	0	1	0
stim 15 min	2.006	0.204	0.761746	0.220015	0.695	0.265
stim 30 min	1.658	0.615	0.612709	0.119174	0.334	0.164
stim 15 min + kinesore	0.970	0.169	1.177639	0.301448	1.263	0.581
stim 30 min + kinesore	1.074	0.144	1.192961	0.363836	0.856	0.262

kif5b densitometry	Trial 1-44	band id	Trial 2-56	band id	Trial 4a-83	band id	Trial 4b-84	band id
unstimulated	825500	5,6 avg	89700	7	219000	1	160000	1
stim 15 min	1530000	1	177000	8	370000	2	401000	2
stim 30 min	1610000	2	10100	9	441000	3	409000	3
stim 15 min + kinesore	823000	3	122000	10	146000	4	137000	4
stim 30 min + kinesore	1020000	4	110000	11	155000	5	180000	5

RMPC-II densitometry	Trial 2-56	band id	Trial 3-60	band id	Trial 4-84	band id
unstimulated	677000	14	2560000	1	1690000	1
stim 15 min	413000	15	3060000	2	811000	2
stim 30 min	267000	16	2060000	3	1080000	3
stim 15 min + kinesore	697000	17	4500000	4	1260000	4
stim 30 min + kinesore	637000	18-20	4890000	5	1230000	5

Slp3 densitometry	Trial 2-56	band id	Trial 3-60	band id	Trial 4-84	band id
unstimulated	501000	1	329000	13	880000	19
stim 15 min	214000	2	403000	14	381000	20
stim 30 min	111000	3	216000	15	109000	21
stim 15 min + kinesore	516000	4	778000	16	346000	22
stim 30 min + kinesore	423000	5	433000	17	359000	23

normalized data

kif5b densitometry	Trial 1-44	band id	Trial 2-56	band id	Trial 4a-83	band id	Trial 4b-84	band id	avg	sem
unstimulated	1	5,6 avg	1	7	1	1	1	1	1	0
stim 15 min	1.853422	1	1.973244	8	1.689498	2	2.50625	2	2.005604	0.204063
stim 30 min	1.950333	2	0.112598	9	2.013699	3	2.55625	3	1.65822	0.615278
stim 15 min + kinesore	0.996972	3	1.360089	10	0.666667	4	0.85625	4	0.969994	0.169262
stim 30 min + kinesore	1.235615	4	1.22631	11	0.707763	5	1.125	5	1.073672	0.143778

RMPC-II densitometry	band id	Trial 2-56	band id	Trial 3-60	band id	Trial 4-84	band id	avg	sem
unstimulated		1	14	1	1	1	1	1	0
stim 15 min		0.610044	15	1.195313	2	0.479882	2	0.761746	0.220015
stim 30 min		0.394387	16	0.804688	3	0.639053	3	0.612709	0.119174
stim 15 min + kinesore		1.029542	17	1.757813	4	0.745562	4	1.177639	0.301448
stim 30 min + kinesore		0.940916	18-20	1.910156	5	0.727811	5	1.192961	0.363836

Slp3 densitometry	band id	Trial 2-56	band id	Trial 3-60	band id	Trial 4-84	band id	avg	sem
unstimulated		1	1	1	13	1	19	1	0
stim 15 min		0.427146	2	1.224924	14	0.432955	20	0.695008	0.264963
stim 30 min		0.221557	3	0.656535	15	0.123864	21	0.333985	0.163722
stim 15 min + kinesore		1.02994	4	2.364742	16	0.393182	22	1.262621	0.580909
stim 30 min + kinesore		0.844311	5	1.316109	17	0.407955	23	0.856125	0.262228

blots

Kif5b

Trial 1

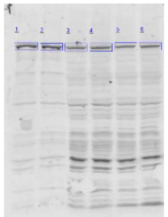

Trial 2

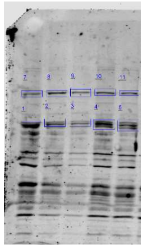

Trial 4a

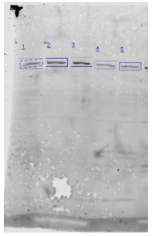

Trial 4b

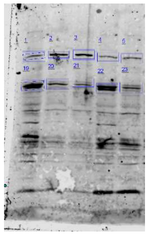

RMCP II

Trial 2

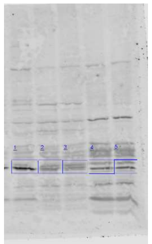

Trial 3

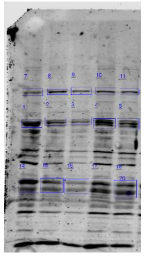

Trial 4

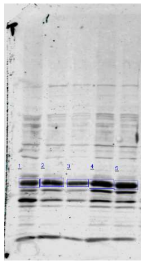

Slp3

Trial 2

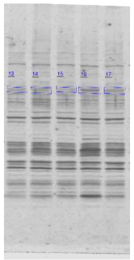

Trial 3

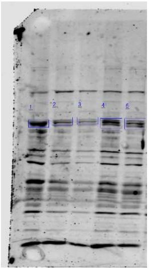

Trial 4

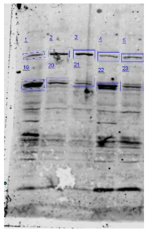

Lysotracker::anti-CD63 labelling co-localization

	Pearsons values (x1000)	
Image	Unstimula	Stimulated
1	0.708	0.601
2	0.76	0.548
3	0.711	0.685
4	0.758	0.659
5	0.683	0.608
6	0.672	0.57
7	0.712	0.684
average	0.714857	0.622143
sem	0.012744	0.020701
ttest		0.002467
